# Mismatching integration-enabled strains and defects engineering in LDH microstructure for high-rate and long-life charge storage

**DOI:** 10.1038/s41467-022-28918-0

**Published:** 2022-03-17

**Authors:** Wei Guo, Chaochao Dun, Chang Yu, Xuedan Song, Feipeng Yang, Wenzheng Kuang, Yuanyang Xie, Shaofeng Li, Zhao Wang, Jinhe Yu, Guosheng Fu, Jinghua Guo, Matthew A. Marcus, Jeffrey J. Urban, Qiuyu Zhang, Jieshan Qiu

**Affiliations:** 1 State Key Lab of Fine Chemicals, School of Chemical Engineering, Liaoning Key Lab for Energy Materials and Chemical Engineering, Dalian University of Technology, Dalian, 116024 China; 2 School of Chemistry and Chemical Engineering, Key Laboratory of Special Functional and Smart Polymer Materials of Ministry of Industry and Information Technology, Northwestern Polytechnical University, Xian, 710072 China; 3 The Molecular Foundry, Lawrence Berkeley National Laboratory, Berkeley, CA 94720 USA; 4 Advanced Light Source, Lawrence Berkeley National Laboratory, Berkeley, CA 94720 USA; 5 Department of Applied and Computational Mathematics and Statistics, University of Notre Dame, Notre Dame, IN 46556 USA; 6 College of Chemical Engineering, Beijing University of Chemical Technology, Beijing, 100029 China

**Keywords:** Two-dimensional materials, Supercapacitors

## Abstract

Layered double hydroxides (LDH) have been extensively investigated for charge storage, however, their development is hampered by the sluggish reaction dynamics. Herein, triggered by mismatching integration of Mn sites, we configured wrinkled Mn/NiCo-LDH with strains and defects, where promoted mass & charge transport behaviors were realized. The well-tailored Mn/NiCo-LDH displays a capacity up to 518 C g^−1^ (1 A g^−1^), a remarkable rate performance (78%@100 A g^−1^) and a long cycle life (without capacity decay after 10,000 cycles). We clarified that the moderate electron transfer between the released Mn species and Co^2+^ serves as the pre-step, while the compressive strain induces structural deformation with promoted reaction dynamics. Theoretical and *operando* investigations further demonstrate that the Mn sites boost ion adsorption/transport and electron transfer, and the Mn-induced effect remains active after multiple charge/discharge processes. This contribution provides some insights for controllable structure design and modulation toward high-efficient energy storage.

## Abstract (web summary)

Layered double hydroxides (LDH) are ideal for charge storage, however, the sluggish reaction dynamics are obstacle to their development. Here, triggered by mismatching integration of Mn sites, the authors configure wrinkled Mn/NiCo-LDH with strains and defects, where promoted mass & charge transport behaviors are realized.

## Introduction

With the gradually growing energy demand and global climate change, the development of electrochemical energy-storage devices for the sustainable energy system has become a research hotspot^1–3^. Among them, the hybrid supercapacitor, which is comprised of battery-like and supercapacitor-like charge-storage processes, grows into a significant type because of the combined (battery-like) high energy density and (supercapacitor-like) high power density^4–6^. To configure high-performance hybrid supercapacitors, it is of importance to modulate & activate the electrochemical reaction properties of the materials. Among the intriguing materials, transition-metal hydroxides, especially layered double hydroxides (LDH) have attracted wide attention over the last decade because of the high theoretical charge-storage capacity, tunable microstructure (e.g. crystal and electronic structure parameters) and superior chemical stability^7–9^. However, the inherent structural properties in nano-/micro-scale always lead to the sluggish reaction dynamics originating from the inhibited ion & electron transport and the insufficient exposure of active sites. It generally results in poor rate capability and stability, which remains a bottleneck for further applications^10,11^. To this end, the development of efficient LDH-based materials requires more innovative investigations in terms of methodologies and design concepts.

According to the thermodynamics and surface chemistry theory, due to the decreasing tendency of overall surface energy, the spontaneous dissolution and migration of the small crystals always happen, further participating in the nucleation of the larger crystals^12–14^. This process is generally accompanied by the transformation from thermodynamically unstable status to stable and highly active status, thus inspiring the configuration of different functional materials with unique merits. For instance, due to the dynamic movement of Ni species from protruding and edge sites to the fringes of large pores, shrinkage of large pores happens for the porous β‐Ni(OH)_2_ skeleton, leading to the generation of highly active β‐Ni(OH)_2_ nanomesh (3–4 nm in size) for promoted reactivity and stability^15^. It was also found that Cu atoms can accomplish the thermodynamically spontaneous insertion in the van der Waals gaps of transition metal dichalcogenides, ultimately promoting the overall electrical conductivity in the z-direction^16^. With the aforementioned information in mind, we predict it could be feasible to advance the microstructure of LDH by the precise incorporation of the spontaneously diffused species during the nucleation process, enabled by thermodynamics-driven downhill of surface energy. On top of that, thanks to the varied atomic radius, coordination status and electronic construction in multiple scales, the incompatibility and local mismatching would happen, resulting in the expansion/compression strain to alter the adjacent atomic arrangement and bonding parameters^17,18^. It can consequently induce irreversible structure regulation or deformation to accommodate abundant lattice distortion and defects, ultimately contributing to the positively modulated reaction dynamics and intrinsic activity^19,20^. To attain this goal, it is highly desired to realize the precise and rational integration of foreign species in LDH microstructure via certain easy-to-implement strategies, and present comprehensive understandings about the modulation effects on the physic-chemical properties.

Herein, we incorporate Mn species from the amorphous MnO_x_ (AMO) into the NiCo-LDH microstructure, successfully configure wrinkled active Mn/NiCo-LDH with built-in strains and defects for enhanced reaction dynamics. The present Mn/NiCo-LDH is ready to be integrated into different substrates, indicative of sound scalability. With the soft X-ray absorption spectroscopy (sXAS) technique, it is found that the released Mn species undergo the strong electron-transfer tendency with Co^2+^, and then participate in the subsequent nucleation reaction, while a moderate reaction concentration is prominent for phase-holding fabrication of the target materials. Guided by finite-element modeling, we demonstrate that the local incompatibility in the LDH matrix-induced compressing strain is robust, thus conferring the efficient incorporation of defects and wrinkles in the tailored topological microstructure. Consequently, the as-configured wrinkled Mn/NiCo-LDH displays a capacity of 518 C g^−1^ (1 A g^−1^), a retention rate of 78% (100 A g^−1^), and a long-cycle life (no performance decay after 10000 cycles). With *operando* Raman characterization, we capture the reversible formation of the oxyhydroxides for Mn/NiCo-LDH, at the same time, we confirm that the Mn-induced effect remains active after multiple charge/discharge processes. Furthermore, density functional theory (DFT) calculation results reveal that the doped Mn contributes to significantly promoted electron-transfer and ion-adsorption capabilities. This work provides some ideas on the design and configuration of LDH-based electrode materials with good charge-storage properties, highlights the insights of the potential mechanisms and structure-property relationship.

## Results

### Configuration route and properties of wrinkled Mn/NiCo-LDH

**Fig. 1:**
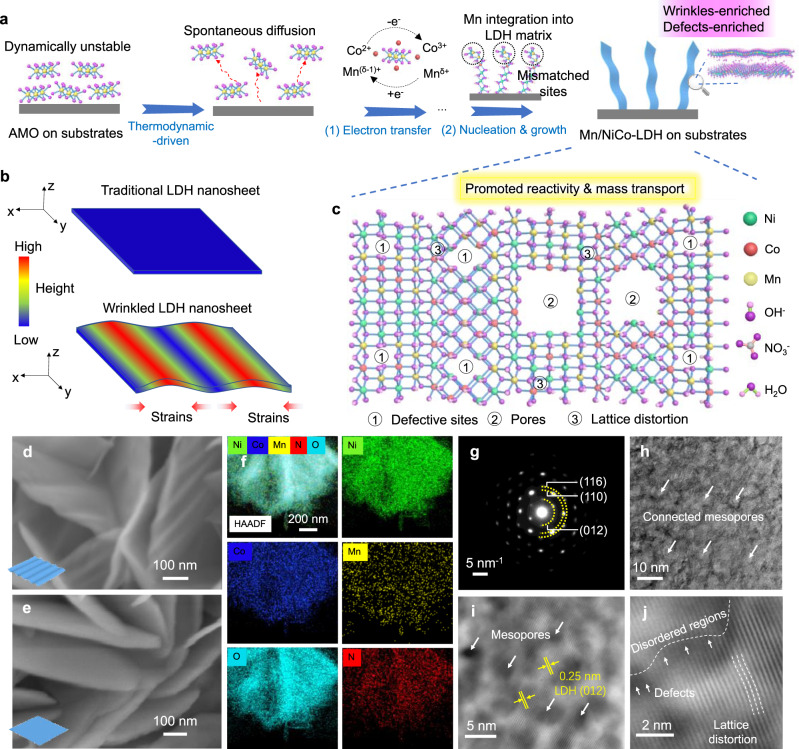
Configuration and microstructure characterization of wrinkled Mn/NiCo-LDH. **a** Formation mechanisms of Mn/NiCo-LDH nanosheets with enriched wrinkles and defects. **b** The comparison of the traditional and wrinkled LDH nanosheets. **c** Schematic illustration for the molecular structure of wrinkled Mn/NiCo-LDH. **d**, **e** SEM images of **d** Mn/NiCo-LDH nanosheets and **e** traditional NiCo-LDH nanosheets. **f** AC-TEM elemental mapping for Mn/NiCo-LDH nanosheets, **g** the corresponding SAED pattern. **h** AC-TEM image of Mn/NiCo-LDH nanosheets. **i**, **j** The as-filtered HAADF-STEM images of Mn/NiCo-LDH nanosheets by combining the origin and inverse-FFT images.

The formation of wrinkled Mn/NiCo-LDH is illustrated in Fig. 1a, where the pre-deposition of the amorphous MnO_x_ (AMO) plays a key role. Specifically, with the high surface energy, AMO demonstrates low thermodynamic stability, thus enabling the spontaneous migration of MnO_x_ motif around the reaction interface. On one hand, these free MnO_x_ motifs undergo irreversible electron transfer with Co^2+^ to produce a certain amount of Co^3+^, which are incorporated into the LDH phase. On the other hand, these motifs display the non-ideal compatibility to the as-fabricated LDH structure, resulting in the generation of considerable strains and defects for the formation of wrinkled nanosheets. For comparison, the traditional flat NiCo-LDH is also configured. As illustrated in Fig. 1b, c, the local incompatibility and lattice mismatching, triggered by the non-ideal integration of Mn sites in multi-scales, can allow the accumulation of strains to tailor defective sites, pores and lattice distortion within the wrinkled microstructure. With these unique merits, the intrinsic reactivity, as well as the mass transport dynamics will be co-promoted, leading to the enhanced charge storage properties.

The microstructures of AMO, NiCo-LDH and Mn/NiCo-LDH were initially investigated by scanning electron microscopy (SEM). The as-deposited AMO demonstrates the small-size nanosheets morphology (Supplementary Fig. 1a). After the mismatching integration-tuned nucleation and growth process, it is interesting to see that the as-formed Mn/NiCo-LDH nanosheets are ultrathin and wrinkled (Fig. 1d). In comparison, the as-formed traditional NiCo-LDH displays a flat nanosheet structure (Fig. 1e). To further explore the microstructures for the as-formed hybrids, transmission electron microscopy (TEM) characterization was then performed. As shown in Supplementary Fig. 2, high-resolution TEM (HR-TEM) images of AMO demonstrate no detectable lattice fringes, indicative of the amorphous microstructure. Besides, it displays a “halo-like” selected-area electron diffraction (SAED) pattern (Supplementary Fig. 1b), further demonstrating its amorphous nature^21,22^. As discussed above, the amorphous feature is favorable to the dynamic migration of free MnO_x_ motifs, contributing to the charge transfer and subsequent mismatching integration & growth process for the generation of Mn/NiCo-LDH with wrinkles. The low-magnification TEM image of Mn/NiCo-LDH displays the wrinkled nanosheet morphology (Supplementary Fig. 3a, b), in accordance with the SEM analysis. The successful incorporation of Mn atoms into the Mn/NiCo-LDH nanosheets is confirmed by the TEM elemental mapping images, which shows considerable Mn Kα fluorescence (Fig. 1f, Supplementary Fig. 3c). As expected, Ni, Co, Mn, N and O are uniformly distributed on Mn/NiCo-LDH nanosheets.

Furthermore, the spherical aberration-corrected TEM (AC-TEM) is adopted for exploring the fine structure. The surface of Mn/NiCo-LDH is found to be exceptionally rough with abundant connected mesopores/voids (Fig. 1h, i), which are responsible for the fast ions transport during the charge-storage process. The enlarged surface area and abundant mesopores are further confirmed by surface area analysis based on Brunauer-Emmett-Teller (BET) theory (Supplementary Fig. 5). Bright and significant diffraction spots can be detected in the SAED pattern, corresponding to the (012), (110) and (116) crystal-planes of the LDH microstructure, respectively (Fig. 1g). The high-angle annular dark-field scanning transmission electron microscopy (HAADF-STEM) images were filtered for better visibility (Fig. 1i, j, Supplementary Fig. 4a–c). The Mn/NiCo-LDH demonstrates the interplanar spacing of about 0.25 nm (Fig. 1i, Supplementary Fig. 4d), corresponding to (012) crystal-plane of the LDH microstructure. Moreover, the microstructure is rich in disordered regions, and endowed with abundant defects as well as lattice distortion (Fig. 1j), which can contribute to the modulated intrinsic reactivity. This spontaneous mismatching-integration route holds considerable potential to controllably and uniformly integrate Mn/NiCo-LDH nanosheets onto different conductive substrates, such as nickel foil, titanium foil, graphite paper, carbon paper, nickel foam and titanium mesh (Supplementary Fig. 6a). As a typical example, a homogeneous deposition of Mn/NiCo-LDH is finally realized on a 100 cm^2^ carbon cloth (CC) (Supplementary Fig. 6b), indicative of the competitive availability and practicality of our reaction route.

**Fig. 2:**
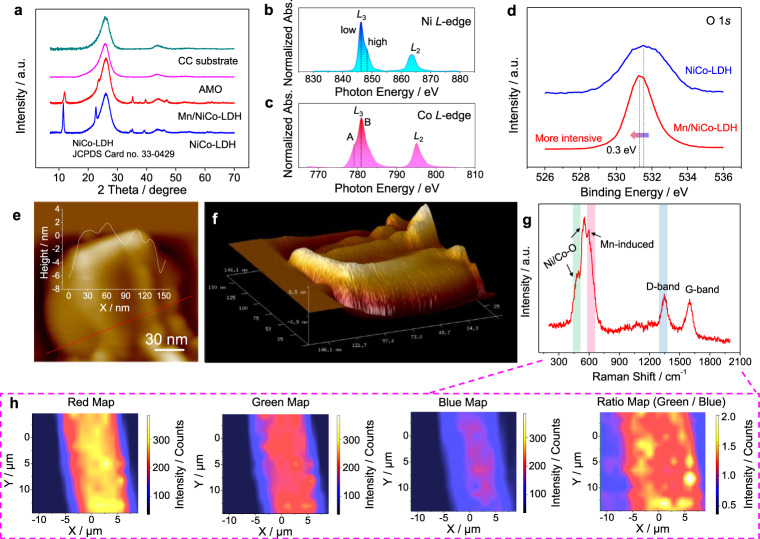
Phase and bonding information analysis. **a** XRD patterns of the pristine CC substrate, as well as the as-formed AMO, Mn/NiCo-LDH and NiCo-LDH; Ni *L*-edge (**b**) and Co *L*-edge (**c**) sXAS spectra of the Mn/NiCo-LDH; **d** O 1 *s* XPS spectra of Mn/NiCo-LDH and NiCo-LDH; AFM 2D (**e**) and 3D (**f**) images of Mn/NiCo-LDH nanosheets, the inset in **e** is the height profit; **g** Raman spectrum of Mn/NiCo-LDH, and **h** the corresponding Raman mapping images.

X-ray diffraction (XRD) patterns of the carbon cloth (CC) substrate, AMO, NiCo-LDH, as well as Mn/NiCo-LDH were studied for further exploring the detailed phase components and crystallization structures. CC substrate displays two typical diffraction peaks at 26° and 43°, corresponding to (002) and (100) planes of the hexagonal graphite-like structure, respectively (Fig. 2a)^23,24^. Besides these two peaks, the as-formed AMO on CC displays no additional diffraction peaks, implying the amorphous structure, agreeing with SAED and HR-TEM results. For the as-formed NiCo-LDH, typical peaks at 11.5, 22.6, 34.4, 35.1, 39.1, 45.9, 60.9 and 62.5° are indexed to (003), (006), (101), (012), (018), (110) and (113) crystal-planes of the typical LDH phase (JCPDS Card no. 33–0429), manifesting its successful formation. It is the same for the as-formed Mn/NiCo-LDH, but the peak position displays the detectable shift to a higher angle. A more detailed analysis was given in Supplementary Fig. 7.

The electronic structure of the Mn/NiCo-LDH is studied by sXAS spectra. Both Ni and Co *L*-edges spectra can be assigned to *L*_3_ and *L*_2_ peaks (Fig. 2b, c), attributed to the spin-orbital coupling interaction. Impressively, the Ni *L*_3_ peak is found to be composed of two peaks with the peak located at lower energy more intensive, indicative of the Ni^2+^-dominated feature^25,26^. The Co *L*_3_-edge spectrum can also be recognized as the sum of two peaks: peak A (low energy, originates from Co^2+^) and peak B (high energy, originates from Co^3+^), respectively. The intensity of peak B is much stronger than peak A (Fig. 2c), implying that most of Co^2+^ are oxidized to Co^3+^ during the nucleation procedure, thanks to the electron transfer with MnO_x_ motifs. With the X-ray photoelectron spectroscopy (XPS) technique, the electronic structures of the Mn/NiCo-LDH were further studied. As shown in Fig. 2d, Mn/NiCo-LDH presents a sharp O 1 *s* intensity as well as the narrow full-width at half-maximum (FWHM) than that of NiCo-LDH (the fitting is given in Supplementary Fig. 15), manifesting that the microstructure is dominated by the bonding of metal-OH with a high phase purity^27,28^. It is worth noting that about 0.3 eV shift to the lower binding energy is found, further elucidating the microstructure modulation enabled by the incorporation of Mn. With the 2D and 3D atomic force microscopy (AFM) images, we also get an intuitive picture of the wrinkled microstructure for Mn/NiCo-LDH nanosheets. It is found that the wave amplitude of the nanosheets is around 1.0–1.5 nm, and the thickness is less than 8 nm (Fig. 2e, f, Supplementary Fig. 8). For NiCo-LDH, it shows the morphology of normal flat nanosheets with a significantly increased thickness (up to 20 nm) as shown in Supplementary Fig. 9. Overall, thanks to the ultrathin and wrinkled structure, Mn/NiCo-LDH holds the abundant ion-permeation channels with shortened diffusion distance, responsible for facilitating the mass-transport kinetics to a great degree.

To gain more information about the bonding form and chemical structure, we subsequently carried out the Raman characterization. For NiCo-LDH, the typical peaks at about 455 and 527 cm^−1^ can be detected (Supplementary Fig. 10), ascribed to bonding of Ni-OH/Co-OH and Ni-O/Co-O^29,30^. For the as-formed Mn/NiCo-LDH, besides these two identified peaks with a slight shift, a new sharp characteristic peak at 605 cm^−1^ emerges (Fig. 2g). Based on the comparisons between the Raman spectrum of Mn/NiCo-LDH, NiCo-LDH, NiMn-LDH, CoMn-LDH as well as numerous standard Mn-based oxides (Supplementary Figs. 10–11), we believe this shoulder peak is likely contributed from the modulated Mn-induced effect, similar to a previous study^31^. The peaks located at about 1395 and 1598 cm^−1^ are attributed to the CC substrate, corresponding to the D- and G- bands^32,33^. The 2D point-by-point Raman mapping reveals a uniform distribution of the typical bonds of Mn/NiCo-LDH with a clear outline of the fiber structure in CC, confirming the homogenous coupling with the substrate without detectable phase separation or aggregation (Fig. 2h) Similarly, the uniform distribution of traditional NiCo-LDH on the substrate is also observed (Supplementary Fig. 11).

### Understanding the fine structure and electronic interaction

**Fig. 3:**
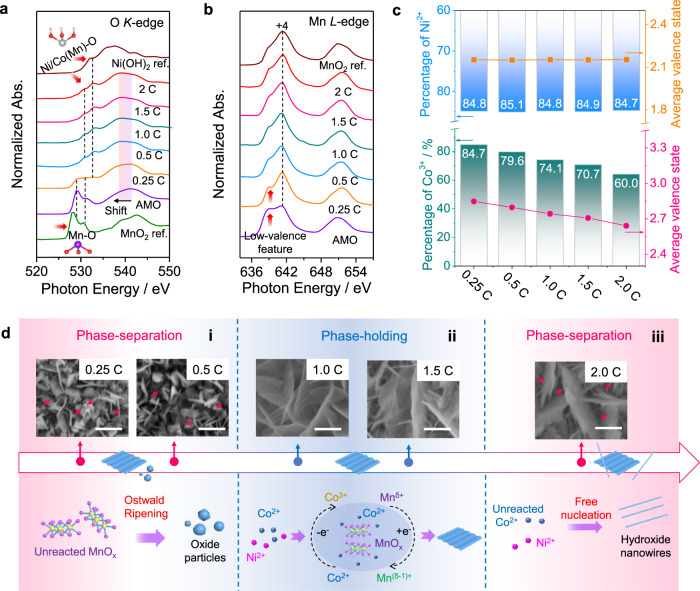
Fine structure and internal electronic interaction. **a** O *K*-edge and **b** Mn *L*-edge spectra of AMO and Mn/NiCo-LDH formed at different reaction concentrations (the reaction concentration for the aforementioned Mn/NiCo-LDH is denoted as 1 C, where C represents the applied reaction concentration.); **c** the variation of valence states for Ni and Co at different reaction concentrations; **d** SEM images of the wrinkled Mn/NiCo-LDH formed at different reaction concentrations and the corresponding reaction mechanism analysis: (i) low concentration (phase-separation); (ii) moderate concentration (phase-holding) and (iii) high concentration (phase-separation). Scale bar, 400 nm.

To further analyze the fine electronic structure and demonstrate the formation mechanisms of the wrinkled structure, reaction concentration-dependent experiments were carried out, and sXAS spectra at the O *K*-edge and Mn *L*_23_-edges were used to understand the fine structure (Fig. 3a, b). Firstly, the pre-edge peaks (from 525 to 535 eV) are analyzed. Notably, in contrast to the MnO_2_ reference sample, AMO also displays two adsorption peaks at around 528 and 531 eV with a small shift (Fig. 3a). This originates from the modulated interaction between Mn 3*d* and O 2*p*. Meanwhile, under a low reaction concentration (a quarter of the adopted concentration, denoted as 0.25 C), besides these typical two peaks, a peak at about 533 eV with a small shoulder peak emerges, corresponding to the covalency of Ni/Co 3*d* and O 2*p*^34,35^. As the reaction concentration increases, the pair of Mn-O peaks from manganese oxides gradually disappear. However, the pair of Ni/Co-O peaks, or more specifically, the Ni/Co(Mn)-O peaks become intensified to some extent, implying that Mn is finely doped in the Ni/Co sites rather than forming a new electronic state in the Mn/NiCo-LDH microstructure. We then focus on the broad peak located from 535 to 545 eV, which is originated from the hybridization between Mn 4*sp* and O 2*p*. Notably, the significant shift toward lower energy is displayed as the reaction concentration increases, ascribed to the decrease of the average valence state of transition metal sites^36,37^. It originates from the generation of the low valent Ni and Co sites besides Mn/NiCo-LDH.

More fine structure information is obtained from the Mn *L*-edge spectra (Fig. 3b). Similar to the MnO_2_ reference sample, the intense peak at about 642 eV can be found for all the as-formed samples, corresponding to the typical feature of Mn^4+^ ^38^. According to the XPS results, the spin energy separations of Mn 3 *s* and Mn 2*p* are calculated to be 4.8 eV and 11.9 eV (Supplementary Fig. 12), respectively, further substantiating the existence of the considerable amount of Mn^4+^ for AMO^39,40^. However, it cannot be ignored that there also exists a distinguishable absorption peak at about 639 eV in the Mn *L*-edge spectrum, implying the formation of Mn sites with lower valence states besides Mn^4+^. Then, the valence states of Co and Ni sites are further investigated by XPS technique. After fitting the Co 2*p*_1/2_ and Ni 2*p*_1/2_ spectra, the percentage of Ni^2+^ and Co^3+^, as well as the average of valence states of Ni and Co sites can be obtained (Fig. 3c and Supplementary Fig. 13). As can be seen, with the decrease of reaction concentration, the percentage of Ni^2+^ displays no significant variation and the average valence states of Ni sites are stable at +2.1. In the case of Co sites, the decrease of reaction concentration yields a significant increase of Co^3+^ and the average valence state.

Accordingly, we can safely conclude that the strong electron transfer of the AMO and Co^2+^ ions (rather than Ni^2+^ ions) exists at the reaction interface, playing a key role during the nucleation process of the Mn/NiCo-LDH nanosheets. With the moderate reaction concentration (1–1.5 C in this case), there are just enough Co^2+^ ions to occupy the appropriate sites for efficient electron transfer with the AMO. This leads to the phase-holding nucleation, thus the uniform wrinkled nanosheets morphology is observed (Fig. 3d(ii)). In fact, phase separation occurs when the reaction concentration becomes either excessive or insufficient. To be specific, with a high reaction concentration, since a certain amount of Co^2+^ cannot undergo the electron transfer with the AMO and thus did not oxidize to Co^3+^, the self-nucleation of the excessive Co^2+^ and Ni^2+^ will happen, leading to the formation of low-valent NiCo-hydroxides nanowires besides the Mn/NiCo-LDH nanosheets (Fig. 3d(iii)). The formation of the new microstructure is also evidenced by the emergence of the new peak at about 12.7° in the XRD patterns as the increase of reaction concentration (Supplementary Fig. 14). While under a low reaction concentration, the free MnO_x_ becomes relatively excessive, resulting in the occurrence of an Ostwald ripening process and the generation of relatively high-crystallinity manganese oxides particles as shown in Fig. 3d(i). This is also evidenced by the results of O 1 *s* XPS spectra. Generally, one can fit the O 1 *s* spectra into four parts: metal-O (O1), metal-OH (O2), surface-adsorbed OH (O3) and surface impurities (O = C-O, O4)^27^. Compared with the high and moderate reaction concentration, the microstructure formed at a low concentration displays a significant increase of O1 (Supplementary Fig. 15), further demonstrating the efficient formation of manganese oxides.

The aforementioned results have revealed that a well-balanced amount of free MnO_x_ and the local reaction concentration is of great significance for phase-holding production of wrinkled Mn/NiCo-LDH nanosheets. To further explore the reaction, we investigate the nucleation process by increasing the amount of AMO at the reaction interface. As expected, with the increased deposition time for AMO, the phase separation, or to say the emergence of the crystal manganese oxide particles, becomes more and more significant based on SEM (Supplementary Fig. 17) and the corresponding XRD analysis (Supplementary Fig. 16).

### Evaluating the charge-storage capability and redox dynamics

**Fig. 4:**
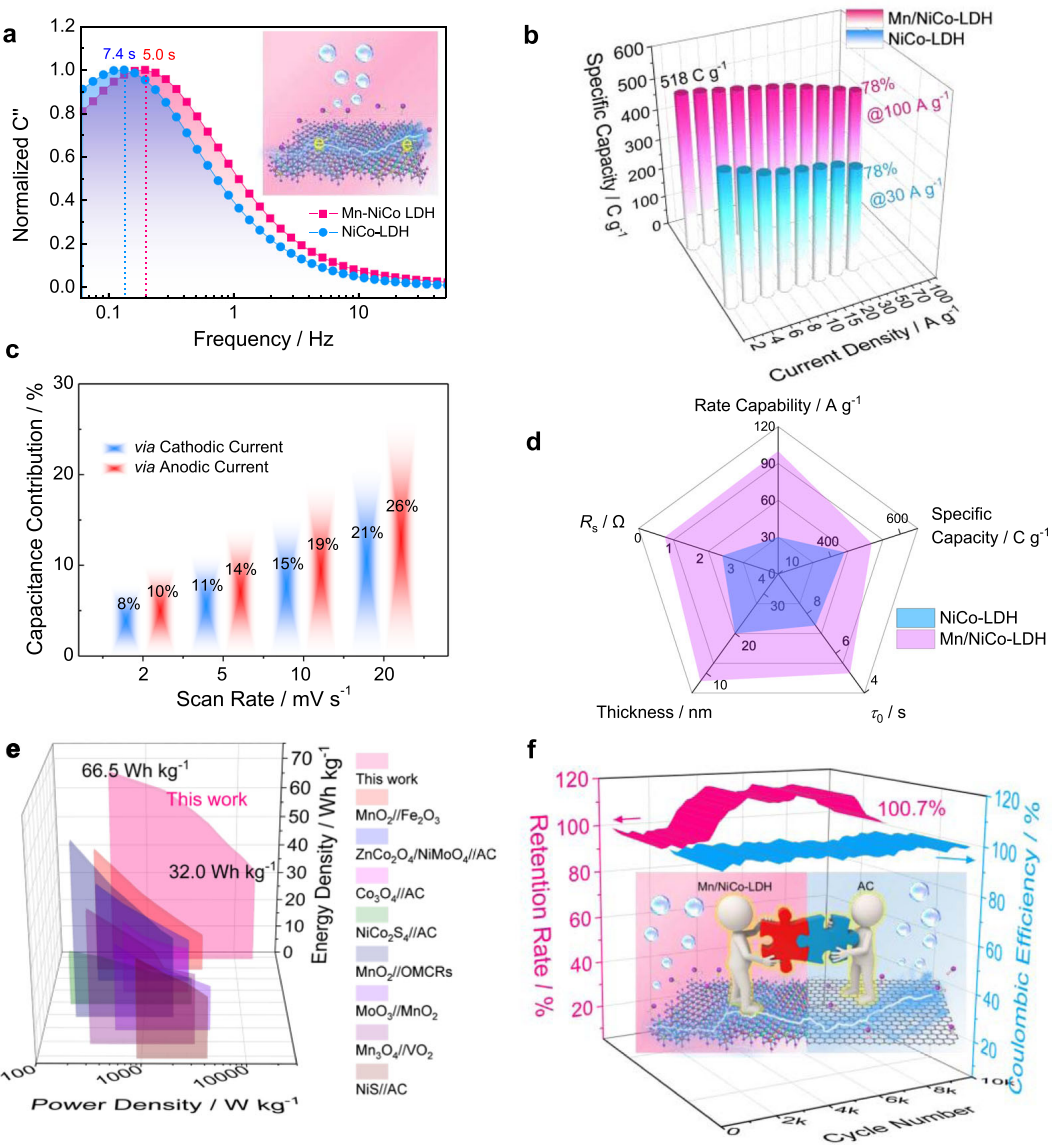
Electrochemical properties of the electrode and asymmetric device in aqueous electrolyte. **a** Relationship between the normalized imaginary part capacitance (*C*”) and frequency for Mn/NiCo-LDH and NiCo-LDH; **b** the specific capacities of Mn/NiCo-LDH and NiCo-LDH; **c** capacitance contributions of Mn/NiCo-LDH; **d** a radar plot comparison of *R*_s_, thickness, *τ*_0_, specific capacity and rate capability for NiCo-LDH and Mn/NiCo-LDH; **e** Ragone plot of the Mn/NiCo-LDH//AC asymmetric device and the comparison with the relative results in literature; **f** cycle performance of the asymmetric device.

Since the novel and unique microstructure features of Mn/NiCo-LDH have been revealed, the electrochemical performance is evaluated. As shown in Supplementary Fig. 18a, the Mn/NiCo-LDH electrode can work well at 50 mV s^−1^, indicative of the sound rate capability. It is further confirmed by the galvanostatic charge/discharge (GCD) curves (Supplementary Fig. 18b), where the reversible charge storage can be realized at 100 A g^−1^. Subsequently, EIS characterizations are performed to investigate the transport behaviors of charge carriers. To the best of our knowledge, the diameter of the semicircle in the high-frequency region of the Nyquist spectrum is generally regarded as the charge-transfer resistance (*R*_ct_). Unlike NiCo-LDH, the Mn/NiCo-LDH electrode demonstrates an extra semicircle at the high-frequency part (Supplementary Fig. 18c), manifesting the existence of more abundant reaction interfaces for enhanced dynamics^41^. Moreover, the semicircle diameter of the Mn/NiCo-LDH electrode is significantly reduced when compared with that of NiCo-LDH, further indicative of reduced charge-transfer resistance. Further, the Mn/NiCo-LDH electrode exhibits the much-shortened relaxation time constant *τ*_0_ (Fig. 4a), thus guaranteeing the easy transport of electrolyte ions^42^. These findings reveal that the finely engineered wrinkled, ultrathin nanosheets have a great promise for high-rate charge storage applications. The capacitance and rate capability of Mn/NiCo-LDH and NiCo-LDH electrodes were studied by GCD curves. As shown in Fig. 4b, the Mn/NiCo-LDH electrode displays a capacity of 518 C g^−1^ (1 A g^−1^), and can achieve a retention rate of 78% (100 A g^−1^) in comparison to the NiCo-LDH electrode (78%@30 A g^−1^).

To have a deep understanding of the charge-storage behavior of Mn/NiCo-LDH, the capacitance contributions are calculated using Dunn’s method (refer to Supplementary Fig. 18 for details). The capacitance contributions at different scan rates are no more than 30% (Fig. 4c), confirming the diffusion-controlled charge-storage properties.

To further identify the advantages of this spontaneous integration route, especially the capability for boosting the energy storage performance, flat NiCoMn-LDH is also fabricated via the direct normal hydrothermal process without pre-deposition of the AMO. It is noted that the as-formed flat NiCoMn-LDH displays a lower specific capacity and rate performance than wrinkled Mn/NiCo-LDH (Supplementary Fig. 19). We thus conclude that the modulation and activation effects of the spontaneous mismatching integration of Mn sites are generated in the LDH microstructure, which favor ion-transport and charge-transfer dynamics across the reaction interface.

With the above information in mind, we show via the radar plot in Fig. 4d that the properties of Mn/NiCo-LDH are much better than those of NiCo-LDH with respect to charge transfer resistance, specific capacity and rate capability. To further test our hypothesis, the asymmetric device is assembled with Mn/NiCo-LDH and the commercial activated carbon (AC), respectively. We find that the device works well at a large operating-voltage window of 1.6 V, displaying no significant deformation of CV curves even at 100 mV s^−1^ (Supplementary Fig. 20a). The reversible charge/discharge can be realized from 0.5 A g^−1^ to 20 A g^−1^, which is illustrated by the high Coulombic efficiency (higher than 90%) and low voltage drop (lower than 100 mV) as demonstrated in Supplementary Fig. 20b, c. Encouragingly, the Mn/NiCo-LDH//AC asymmetric device demonstrates a capacity of 299 C g^−1^ at 1 A g^−1^, and retains 144 C g^−1^ at 20 A g^−1^ (Supplementary Fig. 20d). These results imply the highly enhanced charge storage and good rate capability of the asymmetric device, because of the intriguing microstructure features of Mn/NiCo-LDH as well as the finely matching between Mn/NiCo-LDH and AC. As shown in Fig. 4e, the asymmetric device can exhibit a high energy density of 66.5 Wh kg^−1^ (power density: 0.4 kW kg^−1^), and maintain as high as 32.0 Wh kg^−1^ (power density: 16.0 kW kg^−1^). Remarkably, it outperforms many previously reported results such as MnO_2_//Fe_2_O_3_^43^, ZnCo_2_O_4_/NiMoO_4_//AC^44^, Co_3_O_4_//AC^45^, NiCo_2_S_4_//AC^46^, MnO_2_//OMCRs^47^, MnO_3_//MnO_2_^47^, Mn_3_O_4_//VO_2_^48^ and NiS//AC^49^. More importantly, the Mn/NiCo-LDH//AC asymmetric device can realize superior cycle stability without decay even after 10000 cycles and the Coulombic efficiency constantly stays at about 100% (Fig. 4f). The increase of the capacity at the first 2000 cycles is likely attributed to the gradually tuned electrolyte infiltration and the activation of materials.

### Deciphering structure properties and reaction mechanisms

**Fig. 5:**
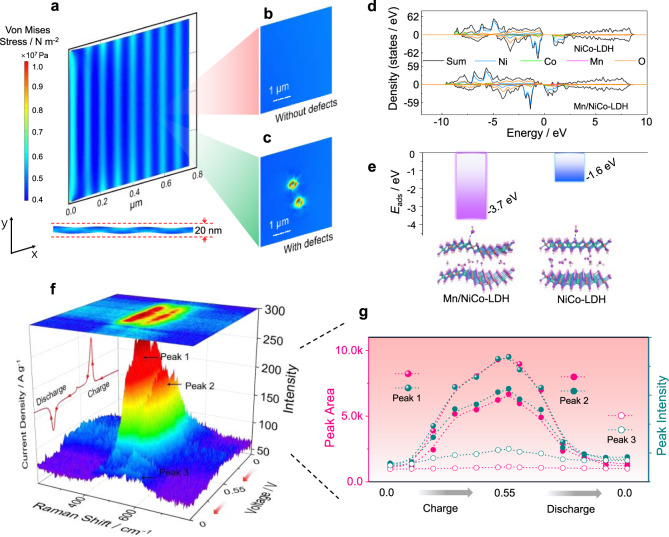
Theoretical calculation and *operando* exploration for understanding the microstructure and reaction mechanisms. **a** The distribution of strain on the nanosheets with the accumulated strain generated by the compressive strain with Mn doping and the constrained force from the substrate. The displacement in the *c*-axis (cross-section) of nanosheets is also given at the bottom. **b**, **c** The effect of the point defects on the distribution of local strain; **d** DOS calculation results of Mn/NiCo-LDH and NiCo-LDH. **e** Adsorption energy toward OH^−^ ions for Mn/NiCo-LDH and NiCo-LDH. **f** *Operando* Raman spectra of Mn/NiCo-LDH during the charging/discharging process (1 mV s^−1^). **g** The change for the typical peak area and intensity in (f), which demonstrates the underlying charge-storage mechanisms.

Besides the superior electrochemical performance, we finally deciphered the intriguing microstructure and reaction mechanisms of Mn/NiCo-LDH. First of all, finite-element analysis was conducted for understanding the origin of the wrinkled nanosheet structure. As depicted in Fig. 5a, the results revealed that the accumulated strain, together with the constrained force (considering the fact that the nanosheets are physically attached on CC), results in wrinkled nanosheets. Moreover, we find out that the appearance of slight amounts of point defects on the nanosheet might also contribute to modulating the distribution of stress, thus responsible for the formation of wrinkled morphology (Fig. 5b, c). Density functional theory (DFT) calculations were then conducted to study the Mn-doping effects on the LDH structure. According to the density of states (DOS) results, we note that Mn doping considerably reduces the bandgap, indicative of the promoted electron-transfer properties. It is also well in accordance with the EIS results. We then evaluate the adsorption energy (*E*_ads_) toward OH^−^ for NiCo-LDH and NiCoMn-LDH. As expected, Mn/NiCo-LDH displays a significantly reduced *E*_ads_ value (−3.7 eV) in comparison with that of NiCo-LDH (−1.6 eV), indicative of the optimization of the active surface with greatly promoted ion adsorption capability. These results afford the deliberate insights on the effects of Mn doping in NiCo-LDH, revealing the concurrent promotion of electron-transfer and ion-transport capabilities in the wrinkled Mn/NiCo-LDH microstructure.

To demonstrate the intrinsic active sites of Mn/NiCo-LDH, *operando* Raman characterization was then conducted (CV scan rate: 1 mV s^−1^). As demonstrated in Fig. 5f and Supplementary Fig. 21, there are three typical peaks located at about 466 (peak 1), 535 (peak 2) and 605 cm^−1^ (peak 3) in the initial state, finely corresponding to the ex-situ Raman spectra (Fig. 2g). First of all, peaks 1 and 2, which are close to the bending mode (*E*_g_) and stretching mode (*A*_1g_) of γ-NiOOH^50–55^, display a continuous increase in peak intensity and area as the charging process proceeds (Fig. 5f, g, Supplementary Fig. 21). In accordance with previous discussions^55–59^, we also ascribed these two peaks to the transformation reaction of NiCo sites from hydroxides to the corresponding (oxy)hydroxides. The intensity of these peaks gradually returns back to the initial status during the discharge process, implying that the transformation process is highly reversible. For the peak at 605 cm^−1^, after careful investigations by considering the internal potential interaction from the adjacent atoms for Mn/NiCo-LDH system^31^, it is likely attributed to the Mn-induced effect with internal interaction. Here, although the variation of peak 3 during the charge/discharge processes is lower when compared with that of peaks 1 and 2, the reversible essential of this Mn-modulated effect is highly reserved. Finally, after multiple *operando* Raman tests under the electrochemical environment (Supplementary Fig. 22), the Raman spectrum displays no obvious change, indicative of the high stability of the Mn-induced effect as well as the reversible formation of the oxyhydroxides for Mn/NiCo-LDH.

Overall, thanks to the mismatching integration of Mn in the microstructure, the superior charge-storage capability of Mn/NiCo-LDH originates from the following aspects: (I) Tuned surface chemistry properties including an ultrathin (<8 nm) and wrinkled nanosheet morphology with mesopores, which are responsible for the efficient exposure of active sites and highly reduced transformation distance of electrolyte ions; (II) Engineered local reaction activity with enriched defective sites, disordered regions and lattice distortion. These unique morphologies result in the redistribution of interfacial charge with high reactivity and are helpful to expose more active sites; (III) Optimized intrinsic redox reaction dynamics: the improved conductivity for fast electron transfer, as well as a reduced adsorption energy barrier toward OH^−^ for fast ion transport.

## Discussion

In summary, based on the mismatching integration of the spontaneous diffused Mn species, we have proposed a promising route to configure wrinkled LDH microstructure with engineered defects and strains. Our sXAS and finite-element modeling results have revealed that the diffused Mn species and Co^2+^ initially undergo strong electron transfer, and then co-participate in the subsequent nucleation, while the in-situ integrated compressive strains govern the generation of the abundant wrinkles. Also, we have demonstrated the reaction concentration should be moderate to achieve phase-holding nucleation. The wrinkled Mn/NiCo-LDH is ready to realize the uniform growth on different conductive substrates, especially the 100 cm^2^ carbon cloth. Ultimately, the as-obtained Mn/NiCo-LDH can achieve a capacity of 518 C g^−1^@1 A g^−1^, and a rate capability of 78% when the current density reaches 100 A g^−1^. In addition, the hybrid supercapacitor, assembled by Mn/NiCo-LDH and commercial activated carbon, can realize an energy density of 66.5 Wh kg^−1^, and display no performance decay after 10000 cycles of testing. Based on the *operando* Raman characterization and DFT calculation results, we have identified that the presented Mn modulation effect features great stability which remains active after multiple charge/discharge processes, at the same time, the integrated Mn sites result in the significant promotion of mass-transport and charge-transfer dynamics for further reducing the reaction barrier. This work serves as the favorable inspiration for positive modulation of LDH microstructure by controllable strains and defects engineering, puts forward some interesting insights to tailor and decouple the intrinsic high-active sites.

## Methods

### Preparation of AMO

AMO was formed by a simple electrodeposition process. Firstly, 245 mg manganese acetate tetrahydrate and 77 mg ammonium acetate were added to a solution of 5 mL dimethyl sulfoxide in 45 mL deionized water. The mixture was stirred for 15 min at room temperature and used as the electrolyte. Then, CC (2 × 3.3 cm^2^) was treated with O_2_ plasma (PCE-6, O_2_ plasma cleaner) for 10 min (power: 29.6 W, pressure: 80 Pa) to yield a superhydrophilic surface. Electrodeposition was conducted on the pre-treated CC, using the saturated calomel electrode and Pt sheet (2 × 2 cm^2^) electrode as the reference and counter electrodes, respectively. The electrodeposition process was conducted on a CHI760E electrochemical workstation at a current density of −2 mA cm^−2^ for 2 min, yielding AMO on CC. After that, the composites were thoroughly washed with deionized water and ethanol, then dried at 60 °C for 2 h.

### Preparation of wrinkled Mn/NiCo-LDH

Nickel(II) nitrate hexahydrate (53 mg), cobalt(II) nitrate hexahydrate (19 mg) and urea (90 mg) were added to 30 mL deionized water under stirring to form a transparent solution. This solution was transferred in a Teflon-lined stainless-steel autoclave (capacity: 50 mL), and AMO (with CC) was then immersed in it. After reaction at 150 °C for 12 h, Mn/NiCo-LDH (with CC) was obtained. Then, the as-formed composites were thoroughly cleaned with deionized water and ethanol, then dried at 60 °C for 2 h. As such, about 1.0 mg cm^−2^ of wrinkled Mn/NiCo-LDH is loaded on CC.

### Material characterizations

The morphology and phase components were examined by XRD (Cu Kα, λ = 1.5406 Å), SEM (HITACHI SU8220), TEM (FEI Talos F200X; Tecnai G2 F30), AC-TEM (FEI Themis Z). XPS (Thermo ESCALAB 250) characterization was carried out to decouple the electronic structure information. Raman mapping and *operando* Raman spectroscopy (HORIBA LabRAM HR Evolution) were conducted to understand the bonding types and underlying charge-storage mechanisms. The laser excitation wavelength is 532 nm, and a laser power of 1–3.2 mW is used to obtain high signal-to-noise spectra without affecting the microstructure. To avoid laser damage, a series of pre-experiments had been done before the *operando* measurement: the sample was dried thoroughly in a vacuum drying oven at 60 °C for eliminating any impurities; ex-situ experiments are carried out for comparison; a pre-measurement was done at a low laser power (less than 0.5 mW), then the power was gradually increased to improve the resolution of typical peaks without altering the local chemical environment. AFM (Bruker, Dimension Icon) characterization was adopted to investigate the surface roughness and wrinkled structure. For fine structure analysis, sXAS spectra were collected in Beamline 7.3.1 of Advanced Light Source (ALS), Lawrence Berkeley National Laboratory (LBNL), and Beamline BL14W1 of Shanghai Synchrotron Radiation Facility (SSRF).

## Supplementary information

Supplementary information

## Data Availability

The data that support the plots within this paper and other finding of this study are available from the corresponding author upon reasonable request.

## References

[1] Li Z (2020). Tuning the interlayer spacing of graphene laminate films for efficient pore utilization towards compact capacitive energy storage. Nat. Energy.

[2] Simon P, Gogotsi Y (2020). Perspectives for electrochemical capacitors and related devices. Nat. Mater..

[3] Murray J, King D (2012). Oil’s tipping point has passed. Nature.

[4] Choi C (2020). Achieving high energy density and high power density with pseudocapacitive materials. Nat. Rev. Mater..

[5] Sun HT (2018). Hierarchical 3D electrodes for electrochemical energy storage. Nat. Rev. Mater..

[6] Dubal DP, Ayyad O, Ruiz V, Gomez-Romero P (2015). Hybrid energy storage: the merging of battery and supercapacitor chemistries. Chem. Soc. Rev..

[7] Sun Y, Terrones M, Schaak RE (2021). Colloidal nanostructures of transition-metal dichalcogenides. Acc. Chem. Res..

[8] Yin H, Tang Z (2016). Ultrathin two-dimensional layered metal hydroxides: an emerging platform for advanced catalysis, energy conversion and storage. Chem. Soc. Rev..

[9] Guo W (2019). A universal converse voltage process for triggering transition metal hybrids in situ phase restruction toward ultrahigh-rate supercapacitors. Adv. Mater..

[10] Li S (2020). Operando revealing dynamic reconstruction of NiCo carbonate hydroxide for high-rate energy storage. Joule.

[11] Guo W, Yu C, Li SF, Qiu JS (2021). Toward commercial-level mass-loading electrodes for supercapacitors: opportunities, challenges and perspectives. Energy Environ. Sci..

[12] Song Y (2017). Ostwald ripening improves rate capability of high mass loading manganese oxide for supercapacitors. ACS Energy Lett..

[13] Liu B, Zeng HC (2005). Symmetric and asymmetric Ostwald ripening in the fabrication of homogeneous core–shell semiconductors. Small.

[14] Li J, Zeng HC (2007). Hollowing Sn-doped TiO_2_ nanospheres via Ostwald ripening. J. Am. Chem. Soc..

[15] Xie J (2017). Intralayered Ostwald ripening to ultrathin nanomesh catalyst with robust oxygen-evolving performance. Adv. Mater..

[16] Liu X-C (2020). Spontaneous self-intercalation of copper atoms into transition metal dichalcogenides. Sci. Adv..

[17] Sun Y (2018). Strong electronic interaction in dual-cation-incorporated NiSe_2_ nanosheets with lattice distortion for highly efficient overall water splitting. Adv. Mater..

[18] Li Z (2021). V “bridged” Co-O to eliminate charge transfer barriers and drive lattice oxygen oxidation during water-splitting. Adv. Funct. Mater..

[19] Gabrys PA (2018). Lattice mismatch in crystalline nanoparticle thin films. Nano Lett..

[20] Gao X (2019). Lattice expansion in optimally doped manganese oxide: an effective structural parameter for enhanced thermochemical water splitting. ACS Catal..

[21] Wu G (2019). A general synthesis approach for amorphous noble metal nanosheets. Nat. Commun..

[22] Zhai P (2021). Engineering single-atomic ruthenium catalytic sites on defective nickel-iron layered double hydroxide for overall water splitting. Nat. Commun..

[23] Chen Q (2018). Yolk–shell NiS_2_ nanoparticle-embedded carbon fibers for flexible fiber-shaped sodium battery. Adv. Energy Mater..

[24] Yu C (2016). NiCo-layered double hydroxides vertically assembled on carbon fiber papers as binder-free high-active electrocatalysts for water oxidation. Carbon.

[25] Yang J (2018). Surface-confined fabrication of ultrathin nickel cobalt-layered double hydroxide nanosheets for high-performance supercapacitors. Adv. Funct. Mater..

[26] Risthaus T (2018). A high-capacity P2 Na_2/3_Ni_1/3_Mn_2/3_O_2_ cathode material for sodium ion batteries with oxygen activity. J. Power Sources.

[27] Wang J (2018). Stabilizing the oxygen vacancies and promoting water-oxidation kinetics in cobalt oxides by lower valence-state doping. Nano Energy.

[28] Yang J (2016). Bridging of ultrathin NiCo_2_O_4_ nanosheets and graphene with polyaniline: a theoretical and experimental study. Chem. Mater..

[29] Yan W, Wang D, Botte GG (2012). Nickel and cobalt bimetallic hydroxide catalysts for urea electro-oxidation. Electrochim. Acta.

[30] Gao M (2022). Nickel-cobalt (oxy)hydroxide battery-type supercapacitor electrode with high mass loading. Chem. Eng. J..

[31] Flores E, Novák P, Aschauer U, Berg EJ (2020). Cation ordering and redox chemistry of layered Ni-rich Li_x_Ni_1–2y_Co_y_Mn_y_O_2_: an operando Raman spectroscopy study. Chem. Mater..

[32] Wang L (2017). Constructing hierarchical tectorum-like α-Fe_2_O_3_/PPy nanoarrays on carbon cloth for solid-state asymmetric supercapacitors. Angew. Chem. Int. Ed..

[33] Li SF (2017). A superhydrophilic “nanoglue” for stabilizing metal hydroxides onto carbon materials for high-energy and ultralong-life asymmetric supercapacitors. Energy Environ. Sci..

[34] Ling T (2018). Atomic-level structure engineering of metal oxides for high-rate oxygen intercalation pseudocapacitance. Sci. Adv..

[35] Li N (2017). Influence of iron doping on tetravalent nickel content in catalytic oxygen evolving films. Proc. Natl Acad. Sci. USA.

[36] Oishi M (2016). Direct observation of reversible oxygen anion redox reaction in Li-rich manganese oxide, Li_2_MnO_3_, studied by soft X-ray absorption spectroscopy. J. Mater. Chem. A.

[37] Guo W (2021). Operando leaching of pre-incorporated Al and mechanism in transition-metal hybrids on carbon substrates for enhanced charge storage. Matter.

[38] Cao X (2021). Stabilizing anionic redox chemistry in a Mn-based layered oxide cathode constructed by Li-deficient pristine state. Adv. Mater..

[39] Wu T-H (2015). Charge storage mechanism of activated manganese oxide composites for pseudocapacitors. J. Mater. Chem. A.

[40] Han G (2014). MnO_2_ nanorods intercalating graphene oxide/polyaniline ternary composites for robust high-performance supercapacitors. Sci. Rep..

[41] Rakhi RB, Alhebshi NA, Anjum DH, Alshareef HN (2014). Nanostructured cobalt sulfide-on-fiber with tunable morphology as electrodes for asymmetric hybrid supercapacitors. J. Mater. Chem. A.

[42] Strauss V, Marsh K, Kowal MD, El-Kady M, Kaner RB (2018). A simple route to porous graphene from carbon nanodots for supercapacitor applications. Adv. Mater..

[43] Tang P-Y (2016). Synergistic effects in 3D honeycomb-like hematite nanoflakes/branched polypyrrole nanoleaves heterostructures as high-performance negative electrodes for asymmetric supercapacitors. Nano Energy.

[44] Hong J (2017). Highly stable 3D porous heterostructures with hierarchically-coordinated octahedral transition metals for enhanced performance supercapacitors. Nano Energy.

[45] Cheng G (2017). O_2_^2−^/O^−^ functionalized oxygen-deficient Co_3_O_4_ nanorods as high performance supercapacitor electrodes and electrocatalysts towards water splitting. Nano Energy.

[46] Xiong X (2015). Controlled synthesis of NiCo_2_S_4_ nanostructured arrays on carbon fiber paper for high-performance pseudocapacitors. Nano Energy.

[47] Shang P, Zhang J, Tang W, Xu Q, Guo S (2016). 2D thin nanoflakes assembled on mesoporous carbon nanorods for enhancing electrocatalysis and for improving asymmetric supercapacitors. Adv. Funct. Mater..

[48] Sahoo R (2018). Redox-driven route for widening voltage window in asymmetric supercapacitor. ACS Nano.

[49] Guan B (2017). Synthesis of hierarchical NiS microflowers for high performance asymmetric supercapacitor. Chem. Eng. J..

[50] Wang Y (2020). Anion etching for accessing rapid and deep self-reconstruction of precatalysts for water oxidation. Matter.

[51] Huang J (2019). Identification of key reversible intermediates in self-reconstructed nickel-based hybrid electrocatalysts for oxygen evolution. Angew. Chem. Int. Ed..

[52] Wu Y (2021). In-situ self-reconstruction of Ni–Fe–Al hybrid phosphides nanosheet arrays enables efficient oxygen evolution in alkaline. Int. J. Hydrog. Energy.

[53] Lee S, Bai L, Hu X (2020). Deciphering iron-dependent activity in oxygen evolution catalyzed by nickel–iron layered double hydroxide. Angew. Chem. Int. Ed..

[54] Lo YL, Hwang BJ (1998). In situ Raman studies on cathodically deposited nickel hydroxide films and electroless Ni−P electrodes in 1 M KOH solution. Langmuir.

[55] Flores E, Vonrüti N, Novák P, Aschauer U, Berg EJ (2018). Elucidation of Li_x_Ni_0.8_Co_0.15_Al_0.05_O_2_ redox chemistry by operando Raman spectroscopy. Chem. Mater..

[56] Yan J (2012). Advanced asymmetric supercapacitors based on Ni(OH)_2_/graphene and porous graphene electrodes with high energy density. Adv. Funct. Mater..

[57] Ji J (2013). Nanoporous Ni(OH)_2_ thin film on 3D ultrathin-graphite foam for asymmetric supercapacitor. ACS Nano.

[58] Guo Y (2019). Multicomponent hierarchical Cu-doped NiCo-LDH/CuO double arrays for ultralong-life hybrid fiber supercapacitor. Adv. Funct. Mater..

[59] Zhao J (2014). Hierarchical NiMn layered double hydroxide/carbon nanotubes architecture with superb energy density for flexible supercapacitors. Adv. Funct. Mater..

