# Supplementary material for: Mismatching integration-enabled strains and defects engineering in LDH microstructure for high-rate and long-life charge storage
Source: Nat Commun. 2022 Mar 17;13:1409. doi: 10.1038/s41467-022-28918-0 (PMC8931012; doi:10.1038/s41467-022-28918-0)
Supplement: Supplementary file 1 — Supplementary information [file 41467_2022_28918_MOESM1_ESM.pdf]

Supplementary information for

Mismatching integration-enabled strains and defects engineering in LDH microstructure for high-rate and long-life charge storage

Guo *et al.*

Table of Contents

Supplementary Methods 3

Supplementary Figures 1-6 5

Supplementary Note 1 11

Supplementary Figures 7-9 12

Supplementary Note 2 15

Supplementary Figures 10-15 16

Supplementary Note 3 22

Supplementary Figures 16-17 23

Supplementary Note 4 25

Supplementary Figure 18 26

Supplementary Note 5 27

Supplementary Figures 19-22 28

Supplementary Table 1 32

Supplementary References 33

Supplementary Methods

Preparation of flat NiCo-LDH. Ethanol acts as the O_2 carrier, due to the high solubility (about 5-7.5 times) of O_2 than that in water. In this case, the dissolved O_2 can enable oxidation of Co^{2+}, thereby leading to coprecipitation of Co^{3+} and Ni^{2+} needed to form NiCo-LDH. Specifically, nickel(II) nitrate hexahydrate (327 mg), cobalt(II) nitrate hexahydrate (109 mg) and cetyltrimethylammonium bromide (500 mg) were put into the solution containing 60 mL ethanol and 12 mL deionized water. The mixture was stirred to form a transparent solution, then poured into a Teflon-lined stainless-steel autoclave (capacity: 100 mL) that contained CC. After reaction at 180 °C for 12 h, flat NiCo-LDH (with CC) was produced. The as-formed composites were thoroughly cleaned with deionized water and ethanol, then dried at 60 °C for 2 h. As such, about 0.9 mg cm^{-2} of flat NiCo-LDH is loaded on CC.

Preparation of flat NiCoMn-LDH. Nickel(II) nitrate hexahydrate (654 mg), cobalt(II) nitrate hexahydrate (218 mg), manganese(II) nitrate solution (50 wt%, 304 mg) and cetyltrimethylammonium bromide (1000 mg) were dissolved in a solution containing ethanol (60 mL) and deionized water (12 mL). The subsequent reaction and treatment procedures are similar to the formation of flat NiCo-LDH. As such, about 1.1 mg cm^{-2} of flat NiCoMn-LDH is loaded on CC.

Electrochemical Characterizations. The electrochemical performance of the as-made materials was initially evaluated in the three-electrode cell with the Hg/HgO electrode, Pt sheet electrode as the reference and counter electrodes, respectively. 6 M KOH aqueous solution was adopted as the electrolyte. The capacity value was evaluated according to the following equation:

$$Q_m=(I\Delta t)/m \quad (1)$$

Here, Q_m represents the specific capacity (C g^{-1}), I represents the current (A), m represents the weight of active materials (g) and Δt represents the discharge time (s).

The energy density (E, Wh kg^{-1}) and power density (P, W kg^{-1}) of the hybrid supercapacitor was evaluated based on the following equation:

$$E=(0.5Q_m\Delta V)/3.6 \quad (2)$$

$$P = 3600E/\Delta t \quad (3)$$

Here, ΔV represents the working voltage window.

Finite-Element Analysis. We initially conduct further analysis on the crystal structure (especially lattice parameters) according to the XRD results. It is revealed that the doping of Mn results in a significant decrease in the lattice parameters along the *a*-, *b*- and c-axis for Mn/NiCo-LDH nanosheets (See Table S1 for more details). Therefore, we predict that the crystal structure with doped Mn exhibits a compressive strain in all directions at the atomic level. Specifically, the compressive strain was determined as 1.3% and 5.1%, respectively along the horizontal and vertical direction of the nanosheet. The computational modeling is performed using the commercial software COMSOL Multiphysics. A model with a nanosheet with a dimensional size of 800 nm × 800 nm was considered, with thickness around 10 nm as obtained from the experimental measurement. The nanosheets were considered as being an elastic material with Young's modulus of 96 GPa and Poisson's ratio of 0.22.[1] A sinusoidal perturbation with an amplitude of 1% of the nanosheet thickness was prescribed to induce buckling of the nanosheets upon compression in the *a*-axis, similar like the previous study.

DFT Calculation. The band structure, projected density of states (PDOS) and adsorption energy calculations were performed in the framework of DFT. All calculations in this paper were performed by using the plane-wave Vienna ab initio Simulation Package[2] (VASP) code with the projector augmented-wave (PAW)[3] pseudopotentials and the PBE[4] exchange-correlation functional. The cutoff energy is set to 520 eV, and the Monkhorst–Pack mesh with 5x5x1 k points is applied for the Brillouin zone integration. The energy convergence criteria for electronic and ionic iterations were 10^{-4} eV and -0.05 eV/Å, respectively. In the LDA+U calculations, we used the value of U=6.6 eV, 6.7 eV for Ni atom, Co atom, respectively. The adsorption energy (E) of OH on the material was defined as follows: $E = E_{slab\text{-}OH} - E_{slab} - E_{OH}$, where $E_{slab\text{-}OH}$, E_{slab}, and E_{OH} correspond to the energies of OH together with slab, the energy of slab and the energy of OH, respectively.

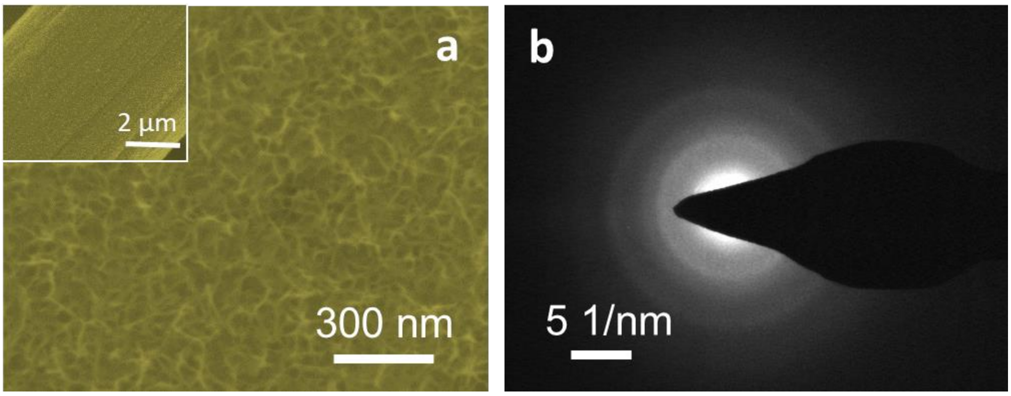

Supplementary Fig. 1 (a) SEM image and (b) SAED pattern of AMO.

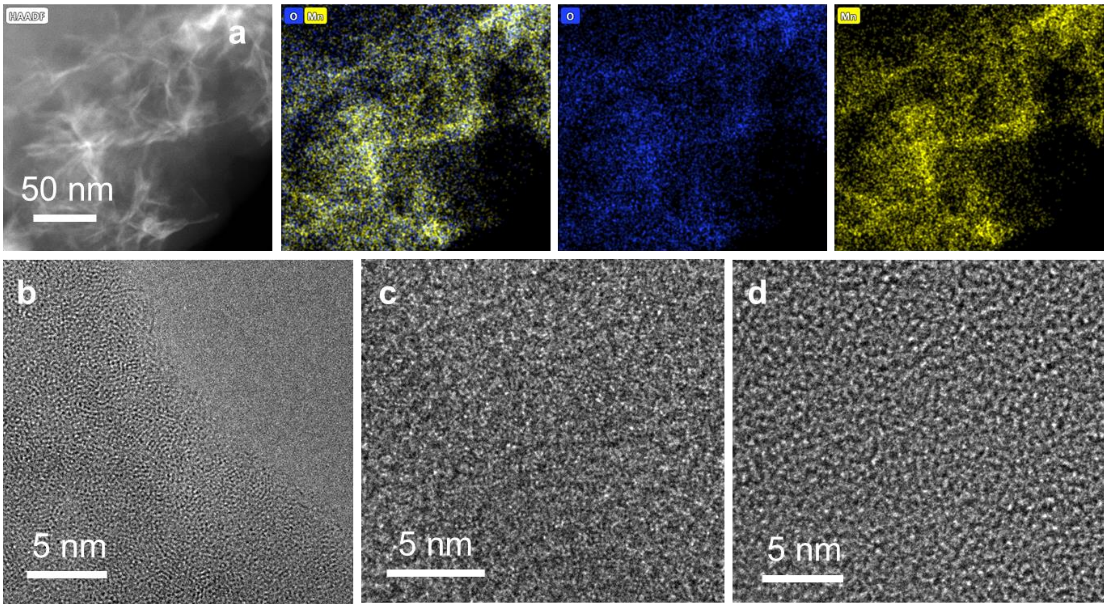

Supplementary Fig. 2 (a) HAADF-STEM image and the corresponding elemental mapping images of AMO. (b-d) HR-TEM images of AMO, suggesting its amorphous feature.

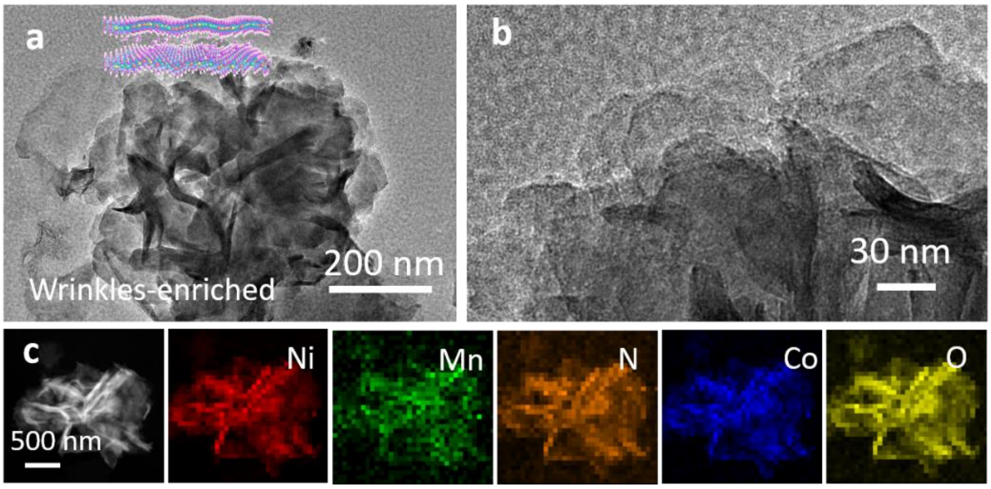

Supplementary Fig. 3 (a-b) TEM images and (c) the corresponding elemental mapping images of Mn/NiCo-LDH nanosheets.

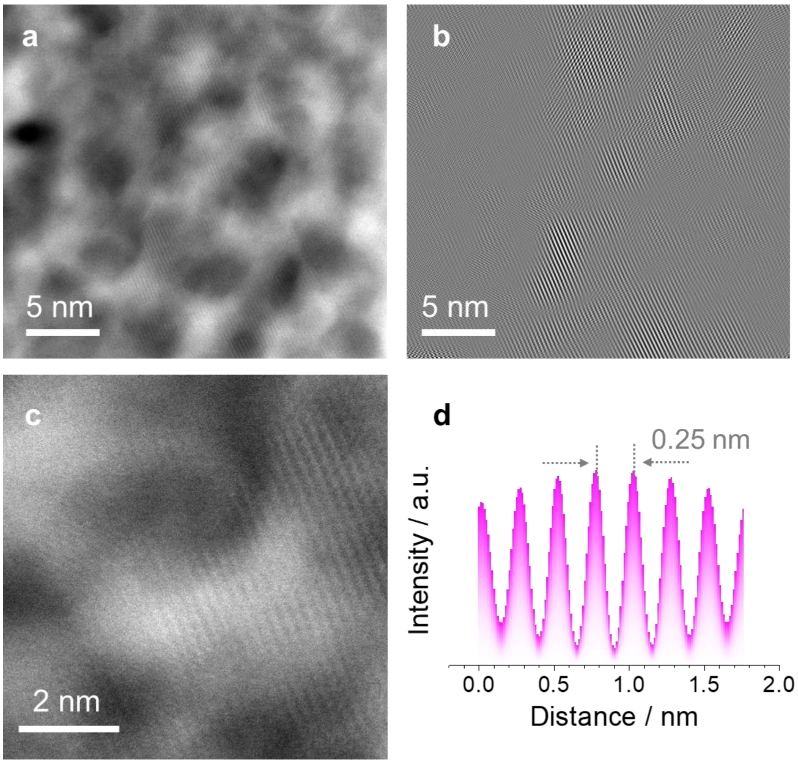

Supplementary Fig. 4 (a, c) HAADF-STEM images of Mn/NiCo-LDH (original images of Fig. 1i and j). (b) the corresponding inverse FFT image of (a), and (d) the lattice distance analysis of Fig. 1i.

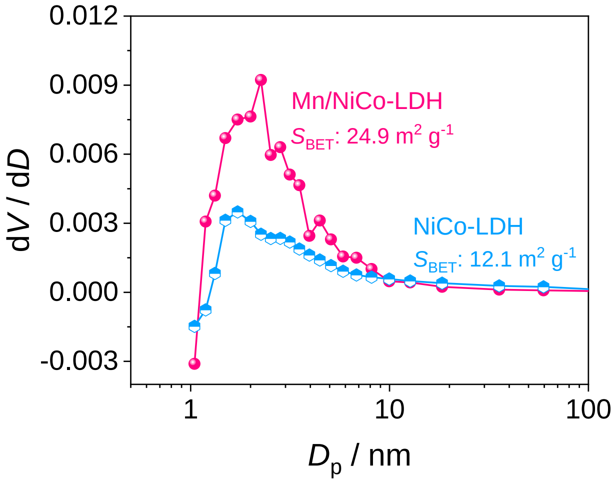

Supplementary Fig. 5 A comparison for the pore size distribution and BET surface area for Mn/NiCo-LDH and NiCo-LDH.

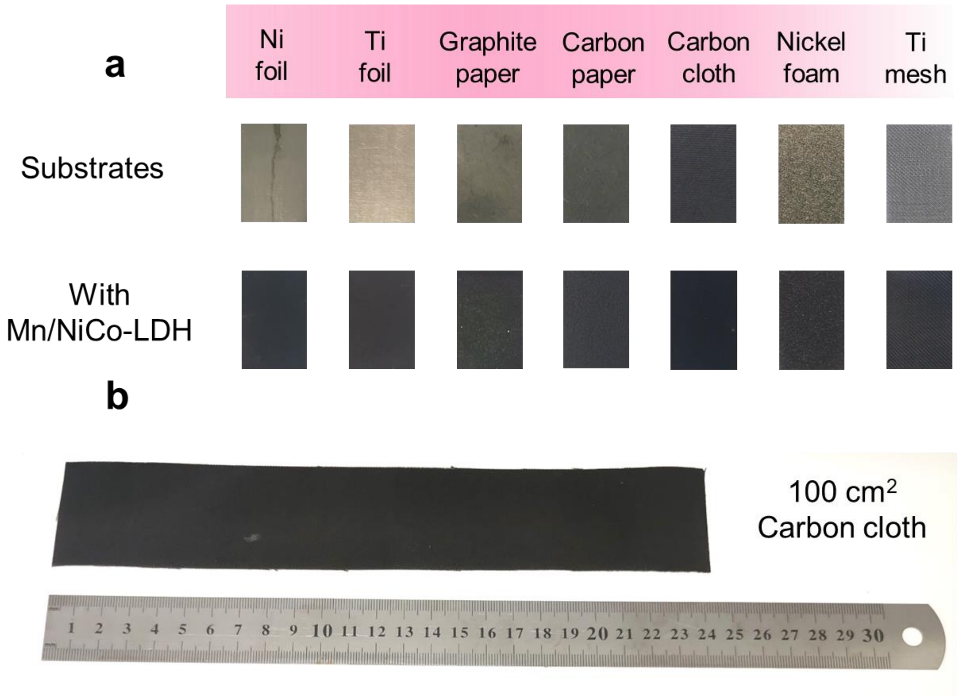

Supplementary Fig. 6 (a) Digital images of various substrates before and after the growth of Mn/NiCo-LDH, (b) digital image of a 100 cm^2 carbon fiber after the deposition of Mn/NiCo-LDH.

Supplementary Note 1

It has been widely accepted that the diffraction angle can indicate the strain-induced expansion and contraction of the lattice.[5-8] Compared with the traditional NiCo-LDH, the XRD pattern of the as-formed Mn/NiCo-LDH displays a slight positive shift to high angles (Supplementary Fig. 7a-b). For example, the peaks corresponding to (012), (015), (018), (003) and (006) planes show the appreciable positive shift of 0.2, 0.5, 0.8, 0.4, 0.8^{o}, respectively, indicative of the existence of compressive strain-induced lattice contraction. As shown in Supplementary Fig. 7c, with the strain effect and modulated interaction, the lattice parameter values of the Mn/NiCo-LDH display a significant decrease when compared with those of standard α-$Ni(OH)_2$ and NiCo-LDH.

On the other hand, the Raman spectrum of Mn/NiCo-LDH is compared with that of the traditional NiCo-LDH. It is found that the related Raman peaks present considerable shift and become broader after the incorporation of Mn (Supplementary Fig. 7d), further indicative of the effect of the internal strain.[9,10]

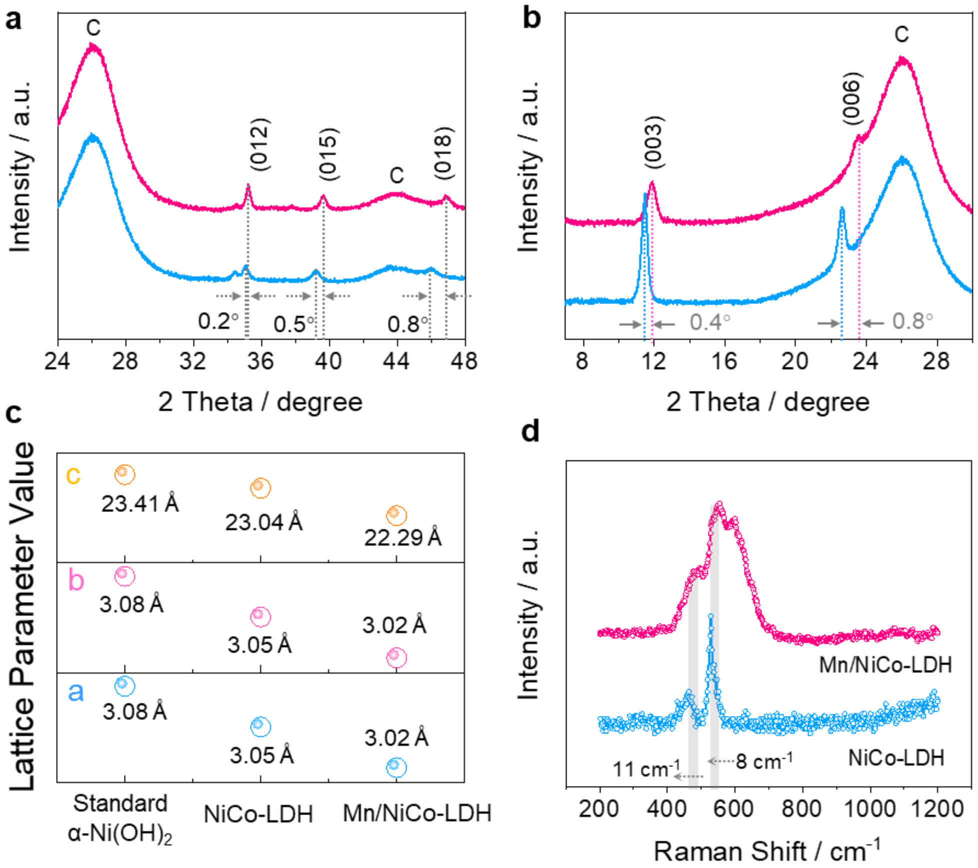

Supplementary Fig. 7 (a-b) A comparison for the magnified XRD patterns of Mn/NiCo-LDH (red) and NiCo-LDH (blue). (c) A comparison for the lattice parameter values of standard α-$Ni(OH)_2$, traditional NiCo-LDH and the as-formed Mn/NiCo-LDH. (d) Raman spectra of the as-formed Mn/NiCo-LDH and traditional NiCo-LDH.

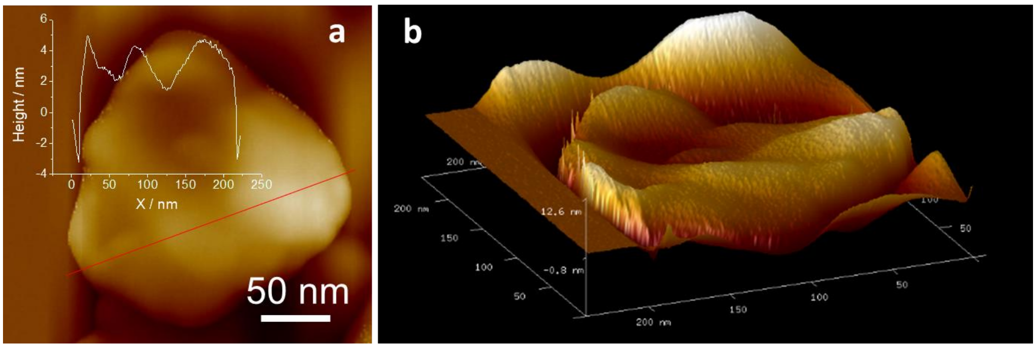

Supplementary Fig. 8 (a) 2D and (b) 3D AFM images of the Mn/NiCo-LDH nanosheets, the inset in (a) is the corresponding height profile.

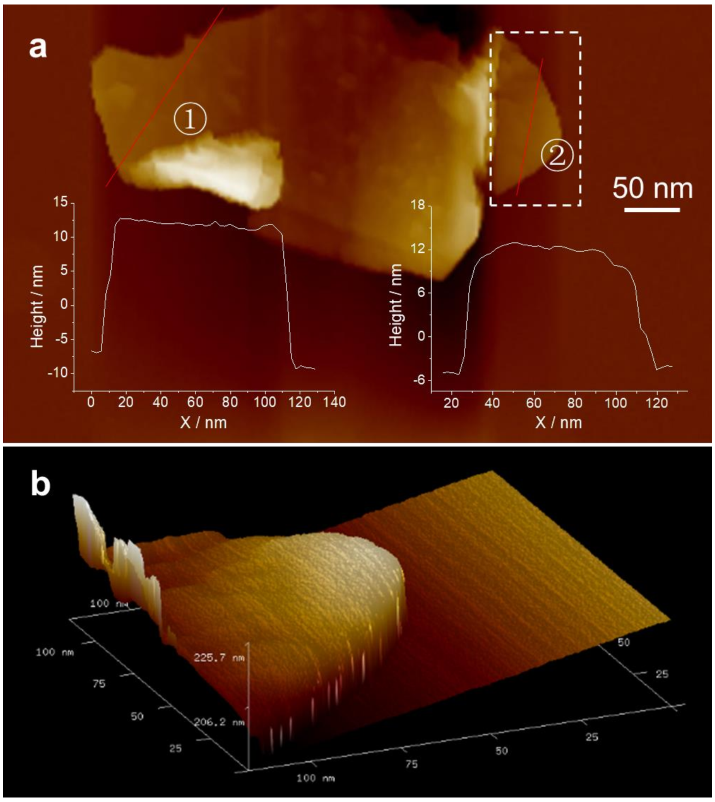

Supplementary Fig. 9 (a) 2D AFM image of the as-formed NiCo-LDH nanosheets, the insets are the corresponding height profits of the region 1 and region 2; (b) 3D AFM image of the NiCo-LDH nanosheets for the region 2 in (a).

Supplementary Note 2

The peak at 605 cm^{-1} is attributed to the Mn-induced effect in the LDH microstructure due to the following considerations:

(1) To have a comprehensive understanding of the Mn-O bond, we have studied the detailed Raman spectra of standard MnO (rock salt structural), Mn_3O_4 (spinel structure), MnO_2 (layered structure) as reference samples, as well as the as-formed Mn/NiCo-LDH in this work (Supplementary Fig. 10). The Raman spectrum of MnO_2 presents three typical peaks at about 501, 561 (basal-plane Mn-O bonds) and 637 cm^{-1} (out-of-plane Mn-O bonds), corresponding well to the layered birnessite-type MnO_2.[11-13] Compared with all Raman features, the shoulder peak at 605 cm^{-1} in Mn/NiCo-LDH does not correspond to any of the Mn-O bonds mentioned above, thus we cannot simply attribute its emergence to the bond of Mn-O.

(2) Since the typical peak at 605 cm^{-1} is not shown for NiCo-LDH (Supplementary Fig. 11), we have further studied more references on the Raman spectra of NiMn-LDH and CoMn-LDH. For NiMn-LDH,[14,15] similar peaks (about 464, 544 and 603 cm^{-1}) like our Mn/NiCo-LDH were found, evidencing that the peak at 605 cm^{-1} originates from the Mn-induced effect. Besides, the Raman spectrum of CoMn-LDH has also been investigated in the related literature,[16,17] which displays the typical peaks at 448 and 530 cm^{-1} as well as the peak at about 605 cm^{-1} (also similar with that of Mn/NiCo-LDH).

The above information implies that this shoulder peak at 605 cm^{-1} should originate from the presence of Mn-induced effect in the LDH microstructure with internal interaction, but not simply from the Mn-O bond.

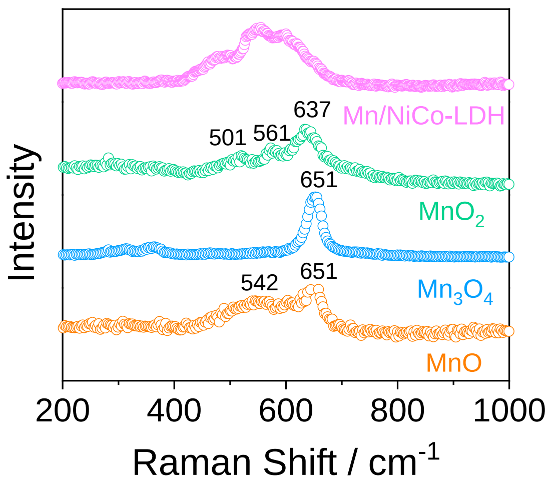

Supplementary Fig. 10 A comparison of Raman spectra among Mn/NiCo-LDH and the reference samples including the standard MnO, Mn_3O_4 and MnO_2.

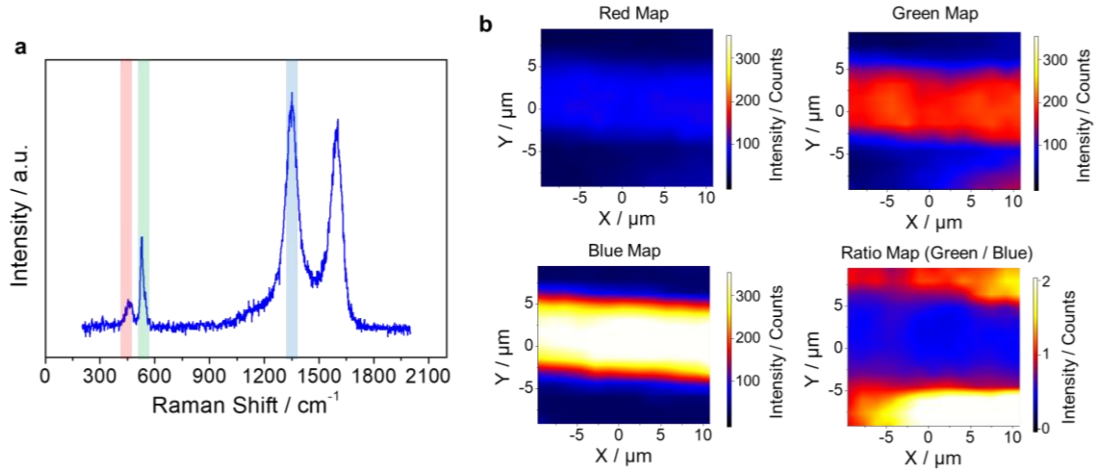

Supplementary Fig. 11 (a) Raman spectrum of the as-formed NiCo-LDH and (b) the corresponding Raman mapping images.

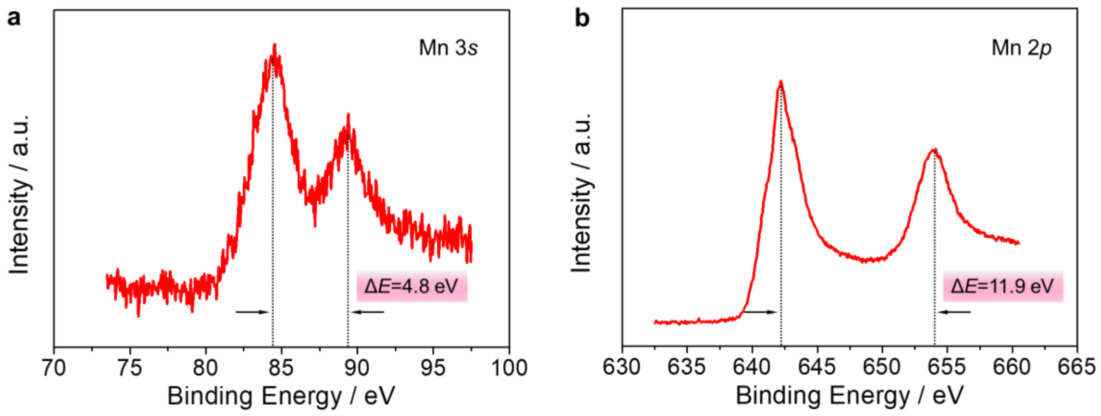

Supplementary Fig. 12 (a) Mn 3*s* and (b) Mn 2*p* XPS spectra of AMO.

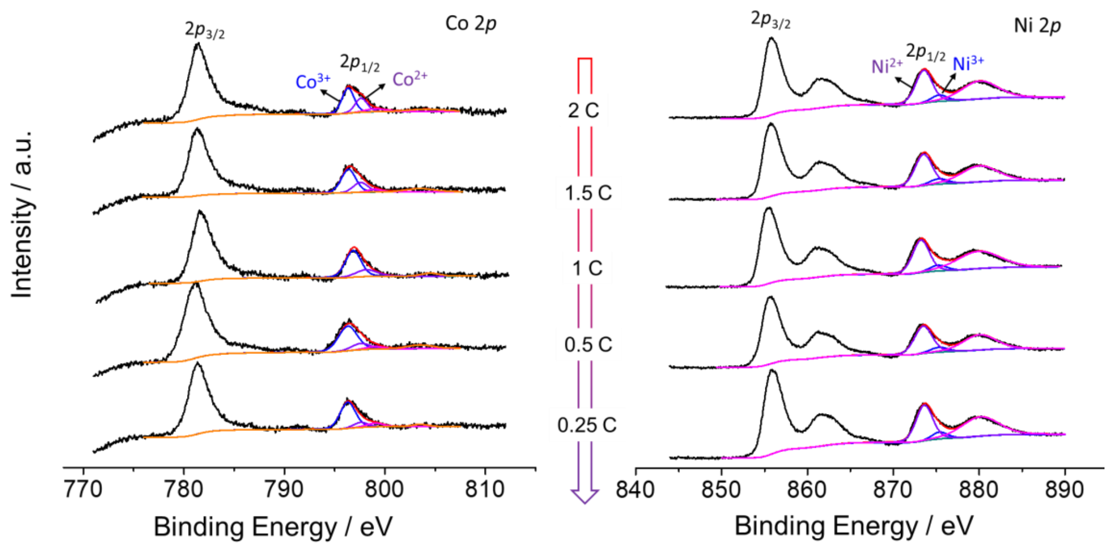

Supplementary Fig. 13 Co 2*p* and Ni 2*p* XPS spectra of Mn/NiCo-LDH formed at different reaction concentrations.

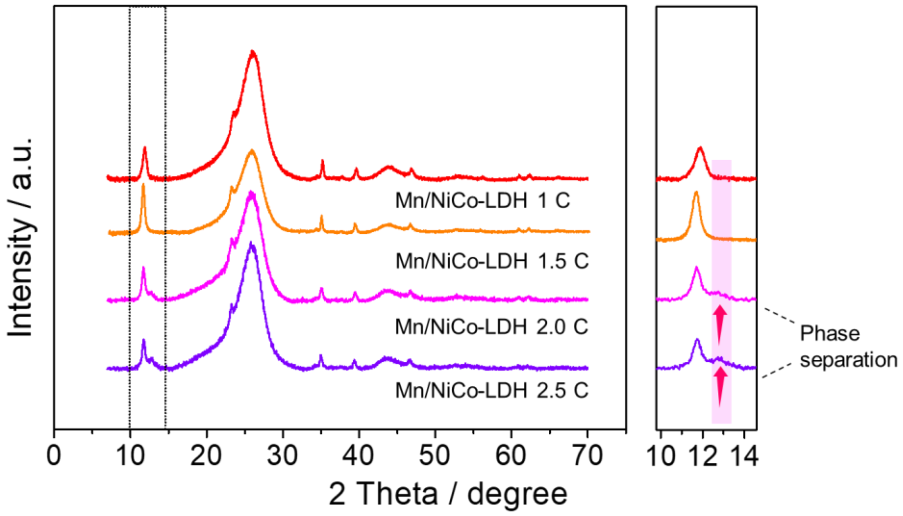

Supplementary Fig. 14 XRD patterns of Mn/NiCo-LDH formed by different reaction concentrations (1 C, 1.5 C, 2.0 C and 2.5 C).

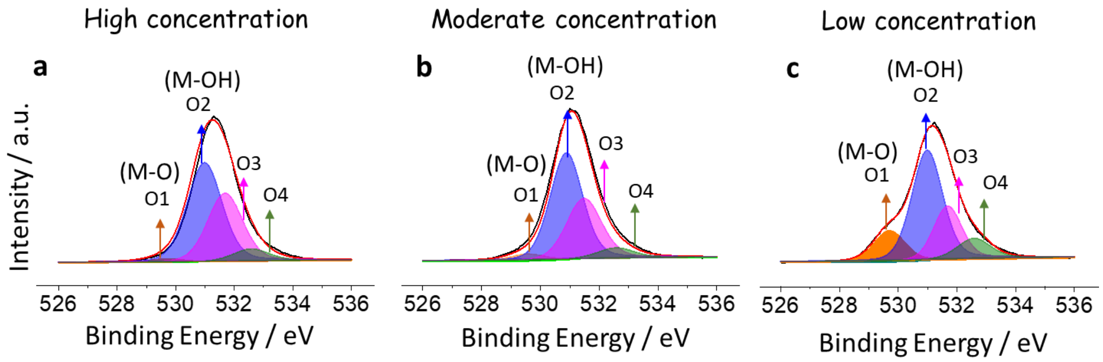

Supplementary Fig. 15 (a-c) O 1*s* XPS spectra comparison of Mn/NiCo-LDH formed at high (2 C), moderate (1 C) and low (0.5 C) reaction concentrations, respectively.

Supplementary Note 3

To gradually increase the amount of diffused free MnO_x at the solid-liquid reaction interface for the nucleation of Mn/NiCo-LDH, the deposition time of AMO was varied from 1 to 15 min. We then carried out the XRD characterization to critically investigate the involved reaction process and the corresponding mechanisms. It is noteworthy that the increase of AMO can result in the generation of new diffraction peaks located at about 19.4^{o} and 36.5^{o}, ascribed to the formation of crystal manganese oxides particles by the Ostwald ripening process (Supplementary Fig. 16). This strongly confirms that the excessive amount of AMO can induce the phase separation growth process, rather than the formation of the pure Mn/NiCo-LDH microstructure. To gain more insights about this, the corresponding microstructure was detected by SEM images. As expected, with the increase of the deposition time of AMO from 5 min to 15 min, the phase separation, or to say the emergence of the crystal manganese oxides particles, become more and more significant (Supplementary Fig. 17), which is in good accordance with the XRD results.

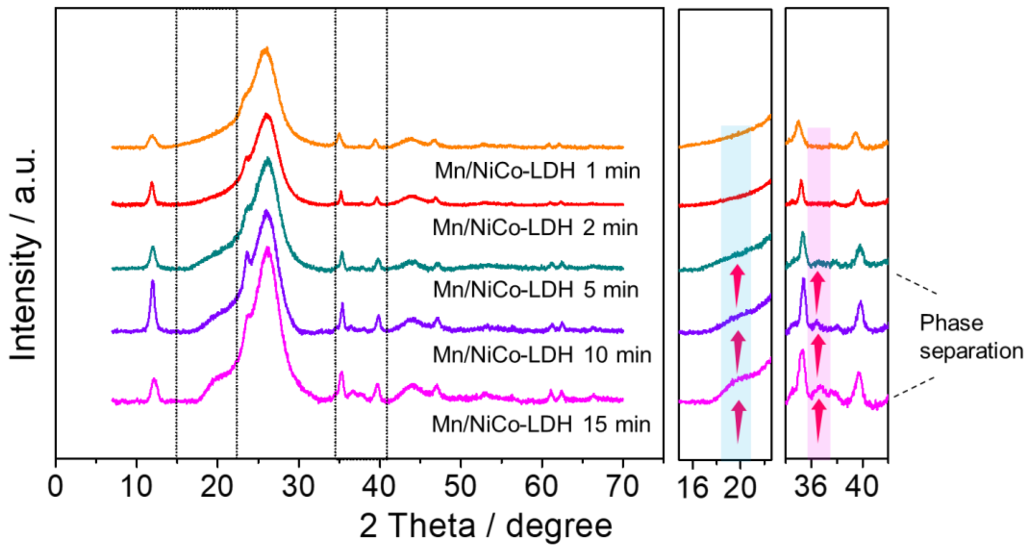

Supplementary Fig. 16 XRD patterns of Mn/NiCo-LDH formed by different electrodeposition times of AMO (1 min, 2 min, 5 min, 10 min and 15 min).

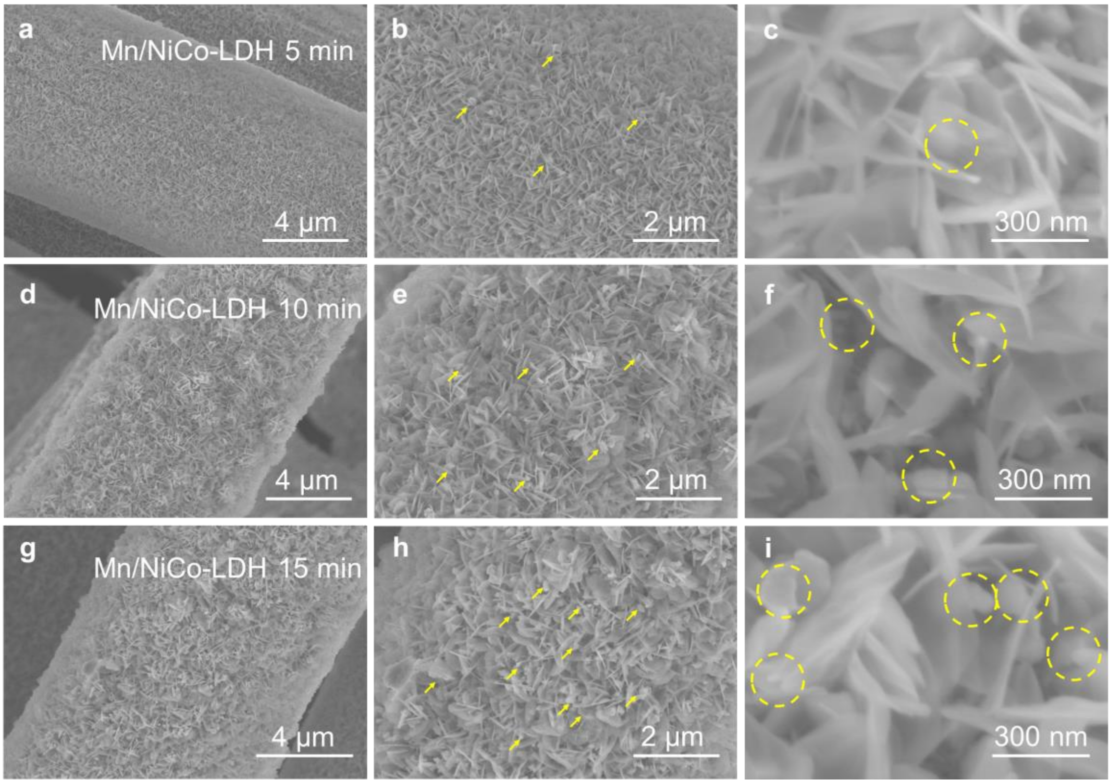

Supplementary Fig. 17 SEM images of Mn/NiCo-LDH formed by different electrodeposition times of AMO: (a-c) 5 min; (d-f) 10 min and (g-i) 15 min (the generated new microstructure is marked by yellow arrows and circles).

Supplementary Note 4.

The redox peak current densities are focused on and further decoupled based on the following equation: $i=kv^b$. Here, i and v represent the peak current density and scan rate, respectively. b is constant, which can suggest the diffusion-controlled (b is close to 0.5) or surface reaction-controlled (b is close to 1) process. As such, a log-log plot of i vs. v should be linear, with a slope given by b. Accordingly, the b values calculated based on the cathodic and anodic peaks are 0.57 and 0.59 (Supplementary Fig. 18d), respectively, indicative of the (battery-like) diffusion-controlled charge-storage behaviors.

Dunn's method is adopted to further clarify the specific capacitive contribution. Specifically, the sum current response (i) is divided into two parts: capacitance contribution current (k_1v) and diffusion-controlled contribution current ($k_2v^{1/2}$), based on the equation: $i=k_1v+k_2v^{1/2}$. This equation can be rearranged as $i/v^{1/2}=k_1v^{1/2}+k_2$, where k_1 and k_2 are constant. It implies that $i/v^{1/2}$ follows the linear relationship with $v^{1/2}$, thus we can determine k_1 and k_2 by plotting the $i/v^{1/2}$ against $v^{1/2}$. Accordingly, the capacitance contribution at different scan rates can be calculated.

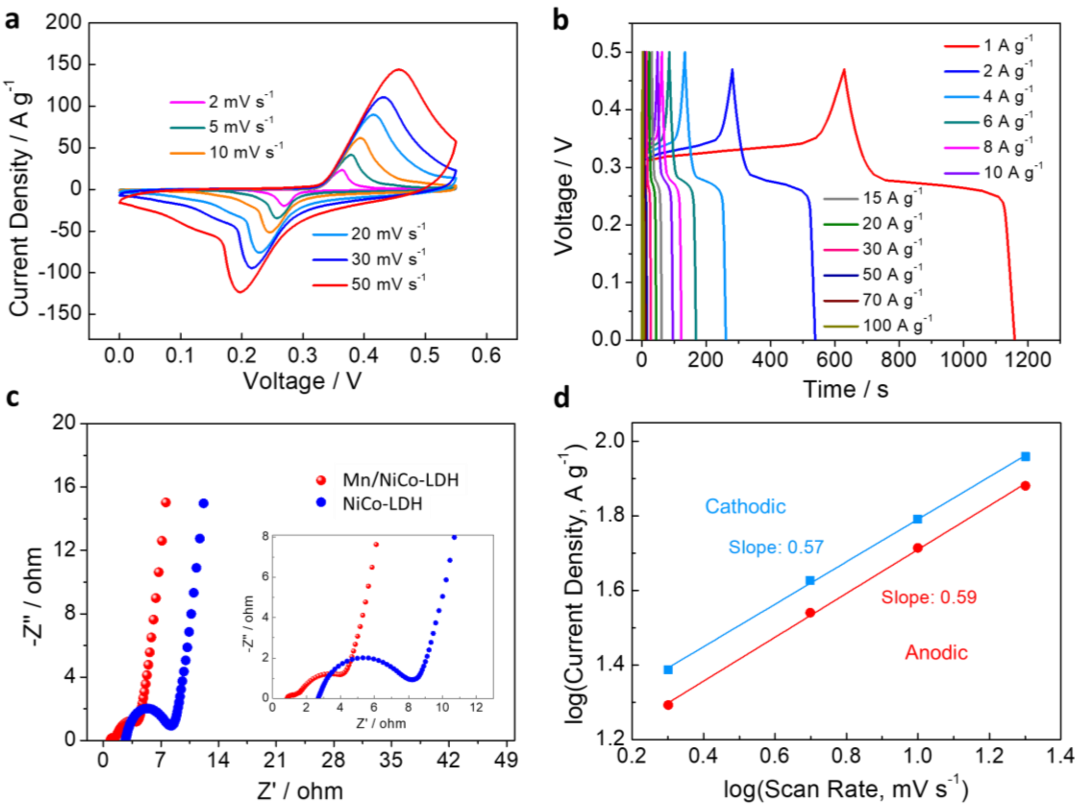

Supplementary Fig. 18 (a) CV and (b) GCD curves of the Mn/NiCo-LDH electrode, (c) EIS spectra of Mn/NiCo-LDH and NiCo-LDH electrodes, (d) the relationship between the logarithm of the peak current density and logarithm of scan rate for the Mn/NiCo-LDH electrode.

Supplementary Note 5

As shown in Supplementary Fig. 19a-b, without the spontaneous mismatching integration effect, the as-prepared NiCoMn-LDH eventually presents the uniform flat nanosheet morphology, instead of the wrinkled microstructure, indicative of the absence of internal strains and incompatibility. The homogeneous distribution of Ni, Co, Mn, N and O elements on the surface is identified by spherical aberration-corrected TEM elemental mapping (Supplementary Fig. 19c). The successful generation of the ternary LDH microstructure by this method is evidenced by the sharp diffraction peaks correlated to LDH phase without distinguishable impurity signals in the XRD pattern (Supplementary Fig. 19d).

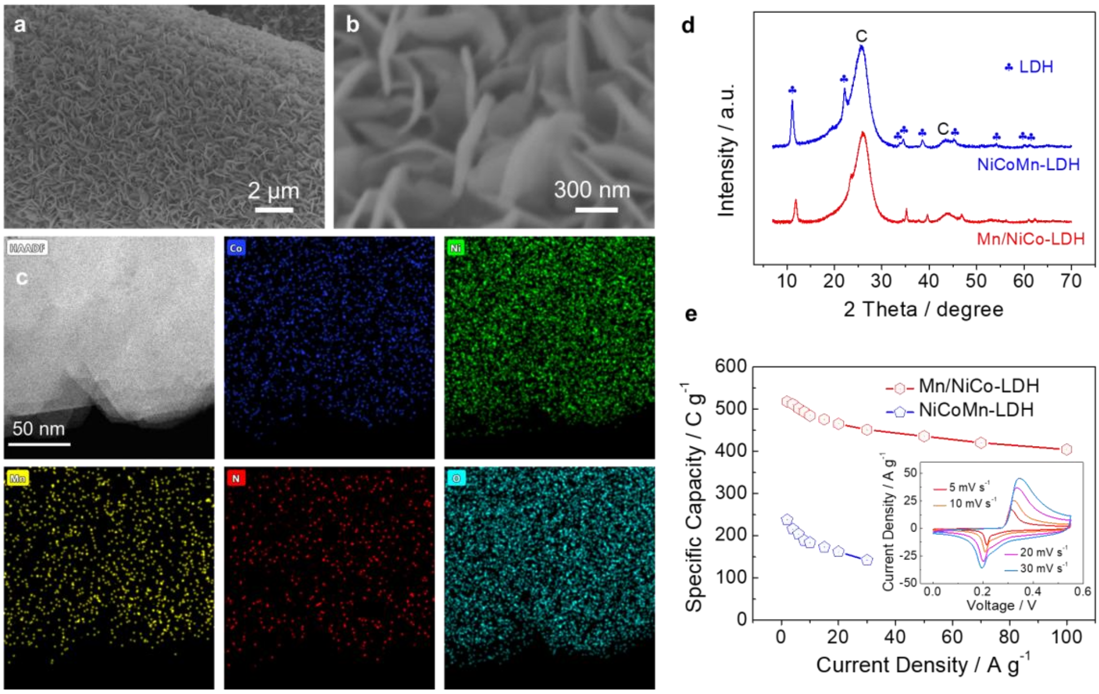

Supplementary Fig. 19 (a-b) SEM images of the as-formed NiCoMn-LDH and (c) the spherical aberration-corrected TEM elemental mapping images; (d) a comparison for XRD patterns of the as-formed NiCoMn-LDH and Mn/NiCo-LDH; (e) specific capacity of Mn/NiCo-LDH and NiCoMn-LDH (the inset is the CV curves of NiCoMn-LDH at different scan rates).

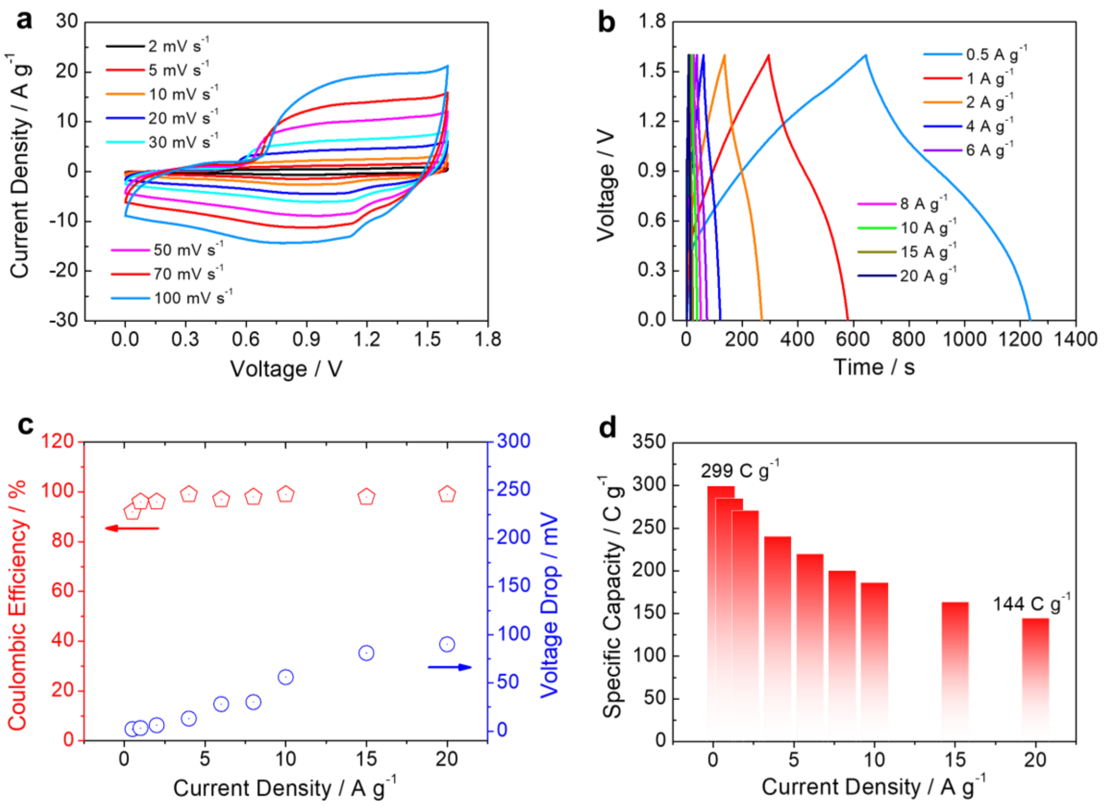

Supplementary Fig. 20 (a) CV curves and (b) GCD curves of the Mn/NiCo-LDH//AC asymmetric device; (c) Coulombic efficiency and voltage drop of the Mn/NiCo-LDH//AC asymmetric device at different current densities; (d) the corresponding specific capacity at different current densities.

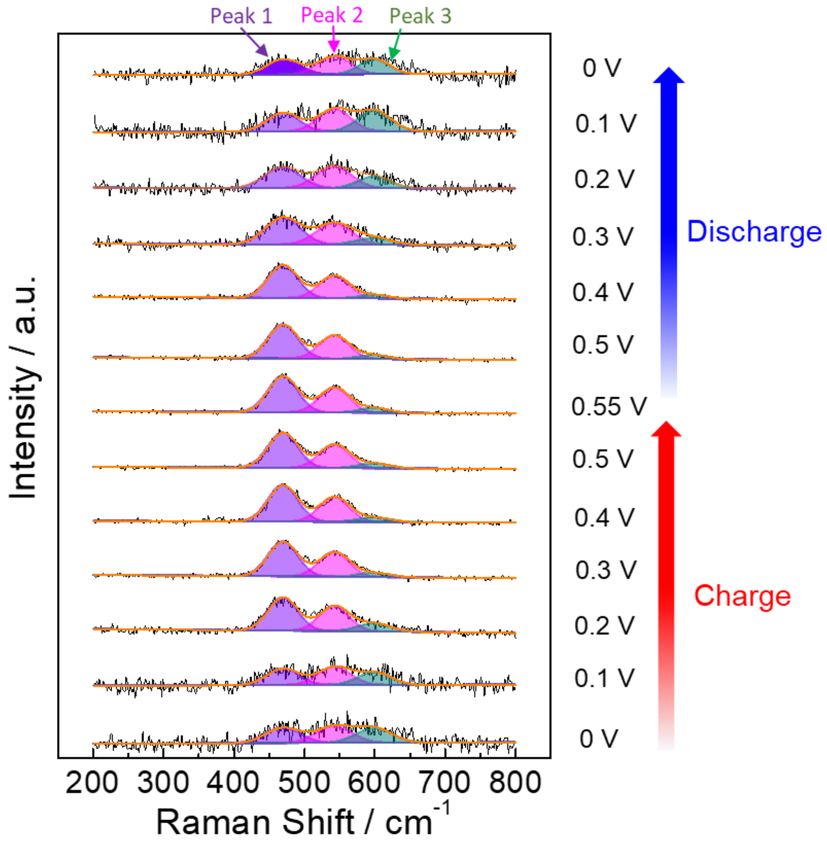

Supplementary Fig. 21 *Operando* Raman spectra of Mn/NiCo-LDH during the charge storage process (CV scan rate: 1 mV s^{-1}).

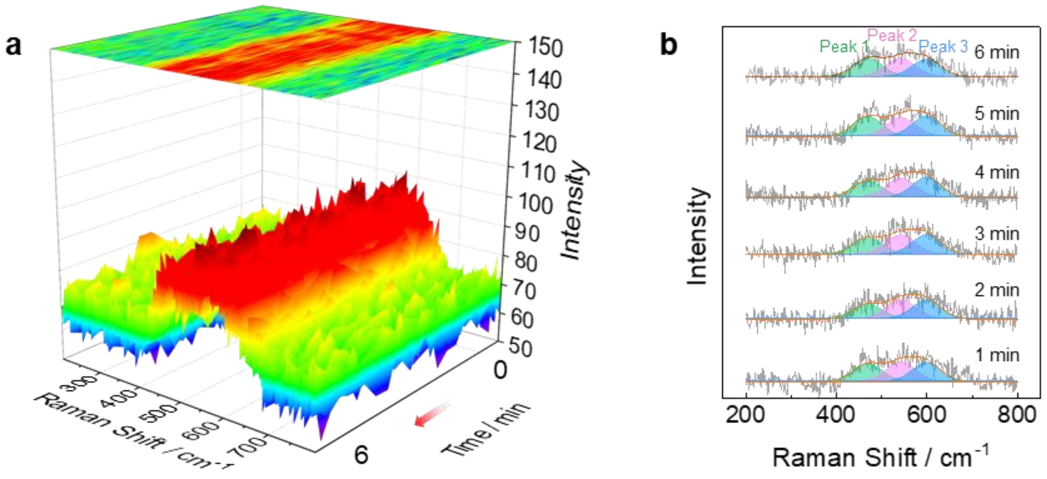

Supplementary Fig. 22 *Operando* Raman spectra (a-b) without external voltage after three electrochemical measurements at 1 mV s^{-1}.

Supplementary Tab. 1 Lattice parameters of the as-fabricated flat NiCo-LDH, wrinkled Mn/NiCo-LDH, and the standard α-$Ni(OH)_2$ (JCPDS Card no. 38-0715).

	**α-$Ni(OH)_2$**	**NiCo-LDH (This work)**	**Mn/NiCo-LDH (This work)**
**α=β**	90°	90°	90°
**γ**	120°	120°	120°
**a=b**	3.08 Å	3.05 Å	3.02 Å
**c**	23.41 Å	23.04 Å	22.29 Å

Supplementary References

1 de Jong, M., Chen, W., Angsten, T., Jain, A., Notestine, R., Gamst, A. *et al.* Charting the complete elastic properties of inorganic crystalline compounds. *Sci. Data* **2**, 150009 (2015).

2 Kresse, G. & Furthmüller, J. Efficient iterative schemes for ab initio total-energy calculations using a plane-wave basis set. *Phys. Rev. B* **54**, 11169-11186 (1996).

3 Blöchl, P. E. Projector augmented-wave method. *Phys. Rev. B* **50**, 17953-17979 (1994).

4 Perdew, J. P., Burke, K. & Ernzerhof, M. Generalized gradient approximation made simple. *Phys. Rev. Lett.* **77**, 3865-3868 (1996).

5 Kim, G., Min, H., Lee Kyoung, S., Lee Do, Y., Yoon So, M. & Seok Sang, I. Impact of strain relaxation on performance of α-formamidinium lead iodide perovskite solar cells. *Science* **370**, 108-112 (2020).

6 Zhou, D., Wang, S., Jia, Y., Xiong, X., Yang, H., Liu, S. *et al.* NiFe hydroxide lattice tensile strain: enhancement of adsorption of oxygenated intermediates for efficient water oxidation catalysis. *Angew. Chem. Int. Ed.* **58**, 736-740 (2019).

7 Dufour, M., Qu, J., Greboval, C., Méthivier, C., Lhuillier, E. & Ithurria, S. Halide ligands to release strain in cadmium chalcogenide nanoplatelets and achieve high brightness. *ACS Nano* **13**, 5326-5334 (2019).

8 Fernandez, A., Caretta, L., Das, S., Klewe, C., Lou, D., Parsonnet, E. *et al.* Strain-induced orbital contributions to oxygen electrocatalysis in transition-metal perovskites. *Adv. Energy Mater.* **11**, 2102175 (2021).

9 Du, Y., Qiu, G., Wang, Y., Si, M., Xu, X., Wu, W. *et al.* One-dimensional van der Waals material tellurium: Raman spectroscopy under strain and magneto-transport. *Nano Lett.* **17**, 3965-3973 (2017).

10 Dadgar, A. M., Scullion, D., Kang, K., Esposito, D., Yang, E. H., Herman, I. P. *et al.* Strain engineering and Raman spectroscopy of monolayer transition metal dichalcogenides. *Chem. Mater.* **30**, 5148-5155 (2018).

11 Boyd, S., Ganeshan, K., Tsai, W.-Y., Wu, T., Saeed, S., Jiang, D.-e. *et al.* Effects

of interlayer confinement and hydration on capacitive charge storage in birnessite. *Nat. Mater.* **20**, 1689-1694 (2021).

12 McKendry, I. G., Mohamad, L. J., Thenuwara, A. C., Marshall, T., Borguet, E., Strongin, D. R. *et al.* Synergistic in-layer cobalt doping and interlayer iron intercalation into layered MnO_2 produces an efficient water oxidation electrocatalyst. *ACS Energy Lett.* **3**, 2280-2285 (2018).

13 Yang, Y., Su, X., Zhang, L., Kerns, P., Achola, L., Hayes, V. *et al.* Intercalating MnO_2 nanosheets with transition metal cations to enhance oxygen evolution. *ChemCatChem* **11**, 1689-1700 (2019).

14 Chala, S. A., Tsai, M.-C., Su, W.-N., Ibrahim, K. B., Thirumalraj, B., Chan, T.-S. *et al.* Hierarchical 3D architectured Ag nanowires shelled with NiMn-layered double hydroxide as an efficient bifunctional oxygen electrocatalyst. *ACS Nano* **14**, 1770-1782 (2020).

15 Zhao, J., Chen, J., Xu, S., Shao, M., Zhang, Q., Wei, F. *et al.* Hierarchical NiMn layered double hydroxide/carbon nanotubes architecture with superb energy density for flexible supercapacitors. *Adv. Funct. Mater.* **24**, 2938-2946 (2014).

16 Zhang, G., Xing, J., Zhao, Y. & Yang, F. Hierarchical N, P co-doped graphene aerogels framework assembling vertically grown CoMn-LDH nanosheets as efficient bifunctional electrocatalyst for rechargeable Zinc-air battery. *J. Colloid Interf. Sci.* **590**, 476-486 (2021).

17 Liu, Z., Yu, C., Han, X., Yang, J., Zhao, C., Huang, H. *et al.* CoMn layered double hydroxides/carbon nanotubes architectures as high-performance electrocatalysts for the oxygen evolution reaction. *ChemElectroChem* **3**, 906-912 (2016).
